# The clinical impacts of lung microbiome in bronchiectasis with fixed airflow obstruction: a prospective cohort study

**DOI:** 10.1186/s12931-024-02931-x

**Published:** 2024-08-14

**Authors:** Yen-Fu Chen, Hsin-Han Hou, Ning Chien, Kai-Zen Lu, Chieh-Hua Lin, Yu-Chieh Liao, Kuo-Lung Lor, Jung-Yien Chien, Chung-Ming Chen, Chung-Yu Chen, Shih-Lung Cheng, Hao-Chien Wang, Po-Ren Hsueh, Chong-Jen Yu

**Affiliations:** 1 Department of Internal Medicine, National Taiwan University Hospital, Yun-Lin Branch, Yunlin County, Taiwan; 2 Graduate Institute of Clinical Medicine, College of Medicine, National Taiwan University, 7 Chung-Shan South Road, Taipei, 100 Taiwan (ROC); 3 Thoracic Medicine Center, Department of Medicine and Surgery, National Taiwan University Hospital Yunlin Branch, Yunlin County, Taiwan; 4 Graduate Institute of Oral Biology, College of Medicine, National Taiwan University, Taipei, Taiwan; 5 Department of Medical Imaging, National Taiwan University Cancer Center, Taipei, Taiwan; 6 Department of Internal Medicine, National Taiwan University Hospital, College of Medicine, National Taiwan University, Taipei, Taiwan; 7 Big Data Center, China Medical University Hospital, Taichung, Taiwan; 8 Institute of Population Health Sciences, National Health Research Institutes, Zhunan, Miaoli 350 Taiwan; 9 Department of Biomedical Engineering, College of Medicine and College of Engineering, National Taiwan University, Taipei, Taiwan; 10 Division of Thoracic Medicine, Far Eastern Memorial Hospital, New Taipei City, Taiwan; 11 Department of Chemical Engineering and Materials Science, Yuan Ze University, Taoyuan City 320, Taiwan; 12 Department of Medicine, National Taiwan University Cancer Center, Taipei, Taiwan; 13 Department of Laboratory Medicine, National Taiwan University Hospital, College of Medicine, National Taiwan University, Taipei, Taiwan; 14 Departments of Laboratory Medicine and Internal Medicine, China Medical University Hospital, Taichung, Taiwan; 15 School of Medicine, China Medical University, Taichung, Taiwan; 16 Ph.D Programme for Aging, College of Medicine, China Medical University, Taichung, Taiwan; 17 Department of Internal Medicine, National Taiwan University Hospital, Hsin-Chu Branch, Hsin-Chu, Taiwan

**Keywords:** Bronchiectasis, Fixed airflow obstruction, COPD, Bronchoalveolar lavage, Lung microbiota, Neutrophilic inflammation, ROSE criteria

## Abstract

**Background:**

Airflow obstruction is a hallmark of disease severity and prognosis in bronchiectasis. The relationship between lung microbiota, airway inflammation, and outcomes in bronchiectasis with fixed airflow obstruction (FAO) remains unclear. This study explores these interactions in bronchiectasis patients, with and without FAO, and compares them to those diagnosed with chronic obstructive pulmonary disease (COPD).

**Methods:**

This prospective observational study in Taiwan enrolled patients with either bronchiectasis or COPD. To analyze the lung microbiome and assess inflammatory markers, bronchoalveolar lavage (BAL) samples were collected for 16S rRNA gene sequencing. The study cohort comprised 181 patients: 86 with COPD, 46 with bronchiectasis, and 49 with bronchiectasis and FAO, as confirmed by spirometry.

**Results:**

Patients with bronchiectasis, with or without FAO, had similar microbiome profiles characterized by reduced alpha diversity and a predominance of *Proteobacteria*, distinctly different from COPD patients who exhibited more *Firmicutes*, greater diversity, and more commensal taxa. Furthermore, compared to COPD and bronchiectasis without FAO, bronchiectasis with FAO showed more severe disease and a higher risk of exacerbations. A significant correlation was found between the presence of *Pseudomonas aeruginosa* and increased airway neutrophilic inflammation such as Interleukin [IL]-1β, IL-8, and tumor necrosis factor-alpha [TNF]-α, as well as with higher bronchiectasis severity, which might contribute to an increased risk of exacerbations. Moreover, in bronchiectasis patients with FAO, the ROSE (Radiology, Obstruction, Symptoms, and Exposure) criteria were employed to classify individuals as either ROSE (+) or ROSE (−), based on smoking history. This classification highlighted differences in clinical features, inflammatory profiles, and slight microbiome variations between ROSE (−) and ROSE (+) patients, suggesting diverse endotypes within the bronchiectasis with FAO group.

**Conclusion:**

Bronchiectasis patients with FAO may exhibit two distinct endotypes, as defined by ROSE criteria, characterized by greater disease severity and a lung microbiome more similar to bronchiectasis without FAO than to COPD. The significant correlation between *Pseudomonas aeruginosa* colonization and increased airway neutrophilic inflammation, as well as disease severity, underscores the clinical relevance of microbial patterns. This finding reinforces the potential role of these patterns in the progression and exacerbations of bronchiectasis with FAO.

**Supplementary Information:**

The online version contains supplementary material available at 10.1186/s12931-024-02931-x.

## Background

Bronchiectasis, a heterogeneous disease in both etiology and clinical presentation [1], results from genetic or acquired conditions [1, 2] and is characterized by permanent airway dilatation and wall thickening [3]. It exhibits diverse radiological and inflammatory patterns, microbiology, patient characteristics, and clinical outcomes [4–6]. Chronic obstructive pulmonary disease (COPD) is a heterogeneous lung condition characterized by persistent airflow obstruction and increased airway inflammatory responses due to prolonged exposure to noxious particles or gases [7]. Patients with bronchiectasis and fixed airflow obstruction (FAO) are those who meet both the obstructive spirometry criteria for COPD and the structural diagnosis of bronchiectasis [8, 9]. The clinical and pathological features coexisting in bronchiectasis and COPD can exacerbate symptoms, intensify inflammation, and worsen prognosis compared to either condition alone [8, 10–15]. A new consensus regarding the definition of “COPD–bronchiectasis association” was proposed by the EMBARC Airway Working Group recently [16]; this definition comprises four components, namely specific **r**adiological signs, functional obstructive pattern, at least two characteristic respiratory symptoms, and current or past smoking (≥ 10 pack-years) or biomass **e**xposure (i.e., ROSE criteria), which are used to describe the coexistence of these two disease entities with complex interactions.

Dysbiosis in the lung microbiome, particularly involving *Proteobacteria* such as *Pseudomonas* and *Haemophilus*, is linked to increased severity and exacerbations in COPD [15, 17–19] and bronchiectasis patients [14, 20, 21]. However, the role of the lung microbiome in bronchiectasis patients with fixed airway obstruction or so-called “bronchiectasis and COPD overlap” remains an under-researched area [13, 15]. A recent study [22] analyzing a United Kingdom cohort used sputum samples to identify five endotypes, revealing distinct inflammatory statuses and microbiological characteristics in COPD, bronchiectasis, and the “COPD-bronchiectasis association” as per the ROSE criteria [16]. This research underscored that traits like neutrophilic inflammation, differential mucin expression, and gram-negative infections are prevalent in patients with the “COPD-bronchiectasis association”. Nevertheless, there is a notable gap in robust data for advanced bronchiectasis patients with fixed airflow obstruction and typical airway symptoms [9], particularly those who do not meet the ROSE criteria due to a lack of smoking history. Additionally, regional variances in etiology, smoking patterns, and environmental exposures in East Asia and other areas may uniquely affect lung microbiology in both COPD [23–25] and bronchiectasis [6, 26]. The complex interplay between the lung microbiome, smoking exposure, and bronchiectasis with airflow obstruction [6, 9, 11, 15, 16, 22] is increasingly recognized. Yet, there is a scarcity of research specifically addressing these relationships within East Asian populations.

In this study, our objective is to investigate the lung microbiome in bronchiectasis patients with FAO using bronchoalveolar lavage (BAL) samples. We aim to evaluate airway inflammatory markers and their clinical relevance, categorizing these patients based on their adherence to the ROSE criteria. Additionally, we will compare these findings with those from patients diagnosed solely with COPD or bronchiectasis within an East Asian cohort.

## Methods

### Study design and participants

Patients with a clinical diagnosis of bronchiectasis or COPD were prospectively recruited between November 2018 and February 2022 from National Taiwan University Hospital (NTUH), Yunlin branch, Yunlin County, Taiwan. We recruited clinically stable patients diagnosed with COPD according to the relevant guidelines [7]. Patients were enrolled if they were aged ≥ 40 years, had a forced expiratory volume in one second (FEV_1_)/forced vital capacity (FVC) ratio < 0.7 at a screening visit, and had a smoking history of at least 10 pack-years or relevant biomass exposure. Bronchiectasis was confirmed by a high-resolution computed tomography (HRCT) scan indicating a bronchoarterial ratio > 1, lack of tapering, and airway visibility within 1 cm of the pleural surface [3, 27], along with clinical symptoms consistent with bronchiectasis. The definition of bronchiectasis with FAO was based on broadly established criteria, encompassing typical airway symptoms (e.g., cough, shortness of breath, wheezing, and sputum production) that met both spirometry criteria for COPD and the structural diagnosis of bronchiectasis [9]. Patients were excluded if they (1) had cystic fibrosis-related bronchiectasis, active allergic bronchopulmonary aspergillosis (ABPA), active pulmonary tuberculosis, or a current diagnosis of asthma; (2) had acute exacerbation of COPD or bronchiectasis within the past 3 months; or (3) were on specific antibiotic treatments or had an acute infection within 1 month prior to the study. Patients on long-term antibiotics or undergoing chemotherapy for malignancy were also excluded.

We collected comprehensive clinical data at enrollment, including outcomes, laboratory and imaging studies, comorbidities, history of exacerbations, current medications, and past major conditions. The definition of exacerbations in COPD and bronchiectasis was based on established guidelines. For COPD, according to the GOLD guidelines [7], a moderate exacerbation requires treatment with antibiotics or systemic glucocorticoids, while a severe exacerbation results in hospitalization or death. In bronchiectasis, an exacerbation episode is characterized by deteriorations in at least three key symptoms within 48 h—such as increased cough, changes in the sputum volume and/or consistency, increased sputum purulence, worsened breathlessness and/or reduced exercise tolerance, fatigue, malaise, and hemoptysis—that necessitate a change in treatment [28]. The severity of exacerbations for both conditions is graded according to the treatment required. Moderate exacerbation episodes necessitate outpatient treatment with antibiotics, systemic glucocorticoids, or other appropriate therapies [7, 28], whereas severe exacerbation episodes require hospitalization or an emergency department visit due to airway complications [7, 28]. The NTUH Research Ethics Committee approved the study (NTUH-REC No. 201712075RINA and 201910082RINA).

### The bronchoalveolar lavage (BAL) samples collection

The participants were asked to fast at least 4 h before undergoing the BAL collection procedure. Participants gargled 20 ml of sterile 0.9% saline (for the collection of oral washing control samples) and then an antiseptic mouthwash containing 0.12% chlorhexidine gluconate immediately before undergoing topical anesthesia and conscious sedation. Before the procedure, 20 ml of sterile 0.9% saline was also washed through the bronchoscope and collected as a control sample. The bronchoscope was inserted into the mouth of a participant and quickly advance d to a wedge position.

In general, with up to 200 ml of 0.9% saline used, BAL fluid was predominantly collected from the right middle lobe in patients with COPD alone in accordance with published protocols [29]. For those with bronchiectasis, BAL fluid was preferentially collected from either the right middle lobe or the left lingual lobe based on the extent of the lobe involvement in bronchiectasis. BAL fluid collection was specifically targeted to the specific lobes with pronounced bronchiectatic changes. If similar levels of severity were noted in multiple lobes, BAL fluid was predominantly collected from the right middle lobe or the left lingual lobe. Although the most affected lobe may not always be the site of sample collection and variability in sampling locations may affect microbiome profiles, we ensured that the selected sites were clinically significant and indicative of active disease. This strategy allowed us to maintain the robustness of our findings while ensuring representative sampling, site accessibility, and patient safety. After the procedure, all the collected samples were sent to our lab within 2 h for subsequent analysis.

### Bronchoalveolar lavage sample for analysis of immune cells, inflammatory cytokines, and neutrophilic extracellular traps

The BAL supernatant was examined for various inflammation markers (e.g., tumor necrosis factor [TNF]-α, interleukin [IL]-1β, IL-6, IL-8, and IL-18) by using a ProcartaPlex Multiplex Immunoassays Kit (Thermo Fisher Scientific) to perform quantitative, multiplexed protein measurements and using Luminex magnetic bead technology per manufacturer recommendations [30]. The collected BAL fluid was filtered through a 40-μm cell strainer (Millipore, Billerica, discarded, and the conidial pellets were resuspended in 200 μl of phosphate-buffered saline (PBS) with the following monoclonal antibodies: CD14, CD15, CD16, CD45, CD49d, CD80, CD206, CD294 (Beckman Coulter), CD163, and CD193 (BioLegend, San Diego, CA, USA). The samples were stained at room temperature in the dark for 30 min and centrifuged at 200×*g* for 5 min. Thereafter, the samples were resuspended in 400 μl of PBS/fix solution (1:1), and a flow cytometric assay (Beckman Coulter) was performed to assess their surface antigen levels [31].

A 96-well plate was coated with myeloperoxidase (MPO) antibodies (1:500) with coating buffer and left overnight at 4 °C. In each well, we replaced the coating buffer with 100 μl of incubation buffer at room temperature for 30 min. Next, in each well, we replaced the incubation buffer with 100 μl of sample buffer at 4 °C, and this condition was maintained overnight. The wells were washed thrice with 300 μl of wash buffer. We added 100 μl of conjugate buffer for neutrophil’s DNA dictation to each well at room temperature for 90 min and then washed each well thrice with 300 μl of wash buffer. Finally, we added 100 μl of substrate buffer at room temperature for 10–20 min and then used an enzyme-linked immunosorbent assay reader for analysis [32].

### Methods for BAL sample processing, DNA extraction, and lung microbiome sequencing

A total of 10 ml of BAL fluid was centrifuged at high speed (13,000 rpm) to pellet cellular material. The bacteria genomic DNA in the BAL samples were extracted using a QIAamp DNA BAL kit (QIAamp DNA Microbiome Kit Cat. No./ID: 51704) according to the manufacturer’s instructions [33]. The bacterial 16S ribosomal RNA variable region V3–V4 was amplified through polymerase chain reaction (PCR) by using the primers V3F (5′-CCTACGGGNGGCWGCAG-3′) and V4R (5′GACTACHVGGGTATCTAATCC-3′) for microbiome analysis (as described in another study [34]) and applying a sample-specific barcode. PCR products were subjected to a microbiome analysis on an Illumina MiSeq sequencing platform with 300-bp paired reads according to the manufacturer’s instructions.

### Lung microbiome analysis

The raw paired-end 16S rRNA sequencing data files were initially analyzed using QIIME 2 with the DADA 2 plugin (version 2022.2) [35] to generate nonchimeric Amplicon sequence variants (ASVs). Taxonomic assignment was performed using the naive Bayesian classifier built-in R package DADA2 (assign Taxonomy function; version 1.22.0) [36] and the curated SILVA 138.1 database (https://github.com/mammerlin/U16S-DD2B/tree/main/Curated%20DB/Curated%20SILVA).

Furthermore, the taxonomy of ASVs assigned as NA (Not Available) at the species level in the DADA2 assignment results was determined using DD2B (https://github.com/mammerlin/U16S-DD2B/tree/main/DD2B) with BLAST + (MGEGABLAST; version 2.12.0) [37].

Finally, the raw ASV abundance was aggregated into the corresponding taxon after taxonomic assignment. The aggregated taxon abundances were then rarified to the minimum number of reads present in the samples for subsequent analyses. For the subsequent data analysis, we use the R software (version 4.1.2) and the Phyloseq [38], vegan [39], microViz [40], and ggplot2 [41] packages. Alpha diversity measurements were calculated using the Shannon index. Beta diversity analysis was performed through a PCoA of Bray–Curtis matrices. Nonparametric statistical analyses, including Wilcoxon rank-sum tests and Kruskal–Wallis tests, were used to compare the relative abundance of taxa and alpha diversity of the groups. Adonis permutational analysis of variance tests were performed to compare the beta diversity between the groups. Pairwise differences in beta diversity were also analyzed by conducting a permutational analysis of multivariate dispersions (Betadisper function in vegan, 999 permutations). Spearman’s correlation test was used to analyze the correlations between clinical variables and selected taxa. Statistical significance was determined using a two-sided *P* value of < 0.05 for diversity analysis or a Benjamini–Hochberg adjusted *P* value of < 0.05 for multiple testing analysis. DESeq2 (version 1.34.0) [42] with “poscounts” size factor estimation and default settings was used to identify differentially abundant taxa between groups of samples. Stacked bar plots of the most abundant taxa were plotted with microViz and ggplot2 packages.

### Negative controls and decomtam method

To address potential background contaminations, we performed DNA extraction and PCR amplification for the biological control (oral washing fluid) and background negative controls (including bronchoscope channel washing fluid, sterile saline and reagents) obtained from study participants from the COPD, bronchiectasis without airflow obstruction (BE), and bronchiectasis with fixed airflow obstruction (BE-FAO) groups in parallel to account for potential contamination. In brief, a total of 78 oral washing control (OWC) samples, 5 bronchoscope channel washing (BCW) fluid samples, 5 sterile normal saline control (NSC), phosphate buffered (PBS) control, 5 extraction kit control (EKC) and 5 non-template control (NTC) were processed for 16S rRNA sequencing.

During the microbiome analysis of 181 BAL samples collected from stable patients, a total of 7771 amplicon sequence variants (ASV) were consolidated to 1750 taxa. To remove the potential contaminations, the combined method in R package Decontam (v1.16.0) [43] with background negative controls were performed and 65 and 20 potential background contaminate species were identified and removed from BAL and OWC samples, resulting in 1685 and 800 species, respectively. Afterward, we performed a rarefaction analysis of 181 BAL samples to obtain the same library size (read count = 20,346). To filter out rare taxa, the remaining 1624 taxa found in fewer than 10% of BAL samples were removed and among ASV annotated to specie, we detected 295 taxa for final analysis in BAL samples (Table S1).

Before removing the contaminants, the BAL and NC showed similar alpha-diversity (Figure S1A), which were significantly higher than OWC samples (*P* < 0.05). The significant differences in the beta diversity (Figure S1B) of microbiome communities among the BAL, OWC and NC samples [R^2^ = 0.331, *P* = 0.01, ADONIS permutational multivariate analysis of variance (PERMANOVA)] were noted.

After the decontam method was performed, microbiome analysis revealed significant differences in alpha diversity between the BAL and OWC samples (P < 0.001, Figure S2A).; and a principal coordinates analysis (PCoA) revealed a significant separation of microbial communities between the BAL and OWC samples (ADONIS PERMANOVA R^2^ = 0.293, *P* = 0.001, Figure S2B), indicating that the microbiome compositions of the BAL and OWC samples were significantly different. However, we still could not exclude the possibility that some lung microbiota of the BAL samples overlapped with pharyngeal taxa because of subclinical microaspiration or the procedural effect [44, 45]. However, no established standards exist for sampling lung microbiome without carry over of upper airway microbes [46, 47], and BAL sampling does present a theoretical risk of exposure to pharyngeal microbiota [47]. Therefore, our procedure protocol for the negative control samples was implemented to minimize background contamination.

### Quantification of emphysema and bronchiectasis severity

All study participants underwent a CT quantification to assess the severity of emphysema. The emphysema severity was quantified by measuring the low-attenuation volume (LAV %), which was segmented at a threshold of -930 Hounsfield units (HUs) relative to the total lung volume on inspiratory CT images [48, 49]. For bronchiectasis, radiological severity was determined using the modified Reiff score, ranging from a maximum of 18 to a minimum of 1, assessing the number of involved lobes [50]. Additionally, the multidimensional bronchiectasis severity index (BSI), which classifies bronchiectasis as mild (0–4), moderate (5–8), or severe (≥ 9), was also evaluated [4].

### Statistical analysis

In this study, continuous variables were presented as mean ± standard deviation (SD) for parametric data and as medians with inter-quartile ranges (IQR) for nonparametric data. For comparing groups, we used the independent samples t-test for parametric data and the Mann–Whitney test for nonparametric data. Categorical variables were analyzed using the chi-square test or Fisher’s exact test, depending on the data suitability. These statistical analyses were conducted using SPSS software (version 18.0, IBM). All tests were two-sided, and a P value of less than 0.05 was considered indicative of statistical significance. The methodology for microbiome analysis and other statistical procedures are detailed as previously described.

## Results

### Clinical characteristics of patients with bronchiectasis and COPD

**Fig. 1:**
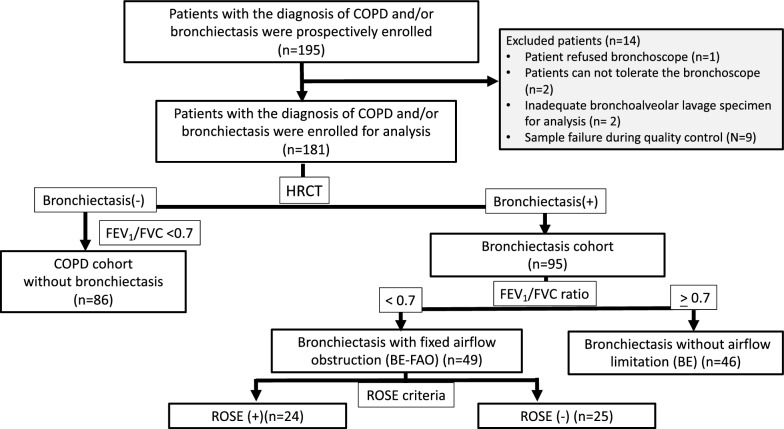
The workflow of patients recruited in the study. *BE* Bronchiectasis without fixed airflow obstruction, *BE-FAO* Bronchiectasis with fixed airflow obstruction, *COPD* Chronic obstructive pulmonary disease, *FEV*_*1*_ forced expiratory volume in 1 s, *FVC* forced vital capacity, *HRCT* high-resolution computed tomography, *ROSE* Radiology, Obstruction, Symptoms, Exposure

Of the 195 consecutively stable patients with COPD and/or bronchiectasis initially enrolled, 181 were included in the final analysis. The study cohort comprised 86 patients with COPD, 46 patients with BE, and 49 patients with BE-FAO (Fig. 1). Their demographic and clinical characteristics are summarized in Table 1. Compared with the COPD group, the BE-FAO group had higher neutrophil counts in the blood and BAL samples, higher C-reactive protein (CRP) levels (Table 2), more exacerbation episodes in the past year, and a higher frequency of prior tuberculosis. Additionally, the BE-FAO group had higher bronchiectasis severity, more extensive emphysema, worse airway symptoms, higher CRP levels, lower lung function indices, and greater reliance on bronchodilators than the BE group.

**Table 1:** Clinical characteristics of study participates (N = 181)

Clinical factors/variables	COPD	BE	BE-FAO	P value BE-FAO vs COPD	P value BE-FAO vs BE
Number	86	46	49		
Age, years, median (IQR)	67.9 (63.1–77.3)	67.1 (59.3–75.4)	73.6 (62.4–78.9)	0.2	0.044*
Gender, Man, n (%)	83 (96.5)	20 (43.5)	35 (71.4)	< 0.001*	0.005*
BMI, median (IQR)	24.2 (22.1–26.2)	21.2 (18.3–24.2)	22.4 (3.8)	0.001*	0.468
Smoking status, n (%)					
Nonsmoker	7 (8.1)	33 (71.7)	25 (51.0)	< 0.001*	0.031*
Ex-smoker or current smoker	79 (91.9)	13 (28.3)	24 (49.0)		
Lung function test, median (IQR)					
FEV_1_/FVC (%)	63.7 (53.1–68.8)	78.0 (74.9–80.0)	64.1 (58.7–66.9)	0.913	< 0.001*
FEV_1_ (%)	73.0 (59.0–85.0)	91.7 (77.7–104.6)	70.0 (53.7–79.8)	0.155	< 0.001*
FVC (%)	91.8 (82.3–107.5)	94.7 (79.3–104.5)	86.4 (73.9–99.6)	0.055	0.11
Bronchodilator reversibility, n (%)	17 (19.8)	4 (8.7)	6 (12.2)	0.191	0.411
Emphysema score, median (IQR)					
LAV < − 930 (HU) (%)	8.5 (2.8–19.9)	2.67 (0.83–8.11)	5.5 (2.7–16.4)	0.224	0.003*
Radiological severity of bronchiectasis					
Bronchiectasis involved lobes, median (IQR)	–	3.0 (2.0–4.0)	4.0 (3.0–5.0)	n.a	0.001*
Modified Reiff score, median (IQR)	–	3.5 (2.0–4.0)	5.0 (3.0–6.0)	n.a	0.010*
Bronchiectasis severity index (BSI), median (IQR)	–	6.0 (3.0–9.0)	8.0 (6.0–10.0)	n.a	0.013*
Mild (0–4), n (%)	–	18(39.1)	6(12.2)	n.a	0.009*
Moderate (5–8), n (%)	–	15(32.6)	20(40.8)		
Severe (≥ 9), n (%)	–	13(28.3)	23(46.9)		
mMRC (dyspnea scale), n (%)					
0–1	50 (58.1)	34 (73.9)	24 (49.0)	0.198	0.011*
≥ 2	36 (41.9)	12 (26.1)	25 (51.0)		
CAT score (symptoms score), n (%)					
< 10	66 (76.7)	33 (71.7)	35 (71.4)	0.314	0.577
≥ 10	20 (23.3)	13 (28.3)	14 (28.6)		
Exacerbation in prior yr					
Low risk: 0–1 time /year	79 (91.9)	38 (82.6)	38 (77.6)	0.020*	0.361
High risk: ≥ 2 times /year	7 (8.1)	8 (17.4)	11 (22.4)		
Comorbidities, n (%)					
Cardiovascular disease	51 (59.3)	15 (32.6)	21 (42.9)	0.048*	0.207
Diabetes mellitus	20 (23.3)	7 (15.2)	5 (10.2)	0.047*	0.335
Chronic kidney disease	13 (15.1)	7 (15.2)	8 (16.3)	0.518	0.554
Chronic liver disease	17 (19.8)	11 (23.9)	6 (13.2)	0.191	0.112
Gastroesophageal reflux disease	53 (61.6)	23 (50.0)	23 (46.9)	0.07	0.463
Obstructive sleep apnea	8 (9.3)	2 (4.3)	4 (8.2)	0.546	0.369
History of tuberculosis infection	3 (3.5)	10 (21.7)	14 (28.6)	< 0.001*	0.229
Autoimmune disease	1 (1.2)	6 (13.0)	2 (4.1)	0.298	0.114
Inhalation therapy, n (%) at baseline					
Short-acting bronchodilator or none	13 (15.1)	34 (73.9)	10 (20.4)	0.214	< 0.001*
Monotherapy (LAMA or LABA)	19 (22.1)	4 (8.7)	4 (8.2)		
Dual therapy (ICS + LABA)	2 (2.3)	0 (0)	0 (0)		
Dual bronchodilators (LAMA + LABA)	38 (44.2)	7 (15.2)	26 (53.1)		
Triple therapy	14 (16.3)	1 (2.2)	9 (18.4)		
Inhaled corticosteroid (ICS)	16 (18.6)	1 (2.2)	9 (18.4)	0.583	0.010*

Data are presented as No. (%) or median (interquartile range), unless otherwise indicated; n.a.: not available

For each row, data are either % with p-values from t test or Fisher’s exact tests between the two groups, median (IQR) with p-values from Mann‐Whitney tests; *P < 0.05

*BE* Bronchiectasis without fixed airflow obstruction, *BE-FAO* Bronchiectasis with fixed airflow obstruction, *BMI* Body Mass Index, *COPD* Chronic obstructive pulmonary disease, *FEV*_*1*_ forced expiratory volume in 1 s, *FVC* forced vital capacity, *LAV* low-attenuation volume, *HU* Hounsfield unit, *CAT* COPD Assessment Test, *mMRC* modified Medical Research Council, *LAMA* long-acting muscarinic antagonist, *LABA* Long-acting β2 Sympathomimetic Agonists, *ICS* Inhaled corticosteroid

**Table 2:** Clinical samples analysis of study patients (N = 181)

Laboratory data	COPD	BE	BE-FAO	P value BE-FAO vs COPD	P value BE-FAO vs BE
Number	86	46	49		
Blood sample, median (IQR)					
Hemoglobin (g/dl)	15.1 (13.6–15.8)	13.5 (12.3–14.2)	13.8 (12.8–14.8)	< 0.001*	0.142
Platelet count (K/μl)	214.5 (183.0–250.2)	228 (193–288)	243 (208–285)	0.004*	0.441
White blood cell counts (K cells/mm^3^)	6.41 (4.97–7.49)	6.64 (4.91–8.22)	7.51 (5.41–9.25)	0.010*	0.058
Neutrophil (%)	59.2 (52.9–64.8)	61.4 (53.3–68.5)	64.4 (57.2–70.8)	0.001*	0.120
Eosinophil (%)	2.8 (1.6–4.3)	2.1 (1.2–3.4)	2.6 (1.4–4.1)	0.263	0.292
< 2%, n (%)	26 (30.2)	22 (47.8)	19 (38.8)	0.205	0.247
≥ 2%, n (%)	60 (69.8)	24 (52.2)	30 (61.2)		
Eosinophil counts (cells/mm^3^)	173.8 (101.1–287.4)	134.7 (65.4–235.3)	162.1 (126.4–271.0)	0.770	0.092
C-reactive protein (mg/dl)	0.15 (0.06–0.31)	0.14 (0.04–0.68)	0.42 (0.23–0.88)	0.001*	0.004*
BAL samples, median (IQR)					
Macrophage %	83.5 (76.3–89.3)	87.1 (77.2–89.6)	85.9 (81.0–91.4)	0.056	0.175
Neutrophils %	1.4 (0.6–2.5)	1.2 (0.7–2.7)	2.1 (0.9–5.2)	0.024*	0.076
Eosinophils %	2.9 (1.8–5.4)	2.3 (1.1–4.3)	1.8 (1.2–2.9)	0.003*	0.290
Lymphocyte %	8.8 (6.4–15.1)	7.4 (3.1–16.6)	8.0 (3.9–10.7)	0.013*	0.655
BAL sample, median (IQR)					
Eotaxin (pg/ml)	1.6 (1.0–5.4)	1.3 (0.7–3.5)	1.9 (0.9–3.5)	0.873	0.375
IL-1β (pg/ml)	4.2 (2.1–9.2)	13.1 (3.7–100.4)	56.0 (4.6–352.6)	< 0.001*	0.095
IL-6 (pg/ml)	10.2 (2.5–21.0)	16.6 (5.4–45.9)	31.1 (6.4–69.7)	< 0.001*	0.175
IL-18 (pg/ml)	31.1 (20.0–52.5)	36.3 (25.4–52.5)	38.3 (23.8–48.9)	0.385	0.754
IL-8 (pg/ml)	201.6 (64.4–377.0)	423.6 (131.0–1453.0)	958.5 (224.8–2616.5)	< 0.001*	0.048*
TNF-α (pg/ml)	4.8 (2.5–9.5)	7.9 (3.9–27.7)	13.2 (5.6–37.7)	< 0.001*	0.074
MCP-1(pg/ml)	171.9 (86.7–311.4)	228.2 (97.7–567.0)	259.5 (165.1–553.4)	0.001*	0.461
NETs (pg/ml)	0.36 (0.18–0.63)	0.57 (0.21–1.04)	1.01 (0.55–2.75)	< 0.001*	0.005*
Conventional culture of BAL samples					
*Klebsiella pneumoniae,* n (%)	14 (30.0)	13 (28.3)	18 (36.7)	0.327	0.255
*Pseudomonas aeroginosa,* n (%)	1 (3.2)	12 (26.1)	16 (32.7)	< 0.001*	0.317
*Staphylococcus aureus,* n (%)	12 (26.1)	4 (8.7)	11 (22.4)	0.436	0.059
*Haemophilus influenzae,* n (%)	6 (13.0)	7 (15.2)	3 (6.1)	0.577	0.134
Non-tuberculosis mycobacterium, n (%)	3 (6.5)	7 (15.2)	7 (14.3)	0.091	0.563
Other bacterial pathogens, n (%)	20 (43.5)	21 (45.7)	20 (40.8)	0.408	0.394
Potential pathogenic bacteria colonization, n (%)	35 (76.1)	38 (82.6)	44 (89.9)	0.008*	0.263
*Aspergillus species,* n (%)	3 (6.5)	3 (6.5)	5 (10.2)	0.543	0.393
*Candida species,* n (%)	5 (10.9)	8 (17.4)	12 (24.5)	0.132	0.276

For each row, data are either % with p-values from t test or Fisher’s exact tests between the two groups, median (interquartile range, IQR) with p-values from Mann‐Whitney tests; *P < 0.05

*BAL* Bronchoalveolar lavage, *BE* Bronchiectasis without fixed airflow obstruction, *BE-FAO* bronchiectasis with fixed airflow obstruction, *COPD* Chronic obstructive pulmonary disease, *IL-1β* interleukin [IL]-1β, *IL-6* interleukin [IL]-6, *IL-8* interleukin [IL]-8, *IL-18* interleukin [IL]-18, *MCP-1* Monocyte chemoattractant protein-1, *NETs* Neutrophil extracellular traps, *TNF-α* tumor necrosis factor [TNF]-α

### Lung microbiome comparison between BE-FAO, BE, and COPD groups

**Fig. 2:**
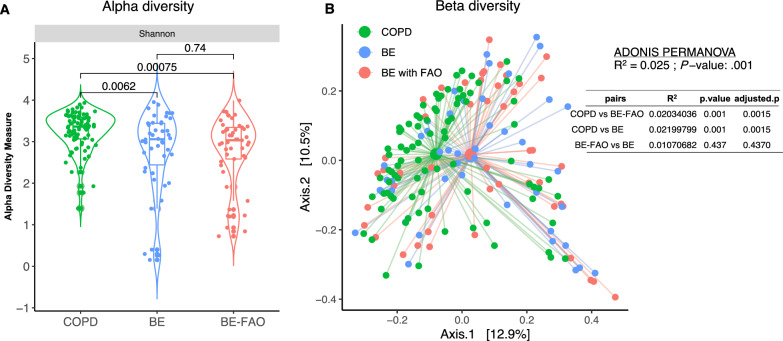
Alpha diversity (**A**) and beta diversity (**B**) of BAL microbiome profiles in three groups. **A** Patients in BE and BE-FAO groups displayed similar Shannon diversity, which were significantly lower than those with COPD alone. **B** The pairwise values using Bray–Curtis distance and principal coordinates analysis (PCoA) to measure the beta diversity between COPD, BE-FAO and BE groups. *BE* Bronchiectasis without fixed airflow obstruction, *BE-FAO* Bronchiectasis with fixed airflow obstruction, *COPD* Chronic obstructive pulmonary disease

In our study, alpha diversity in the BE-FAO and BE groups was significantly lower than that in the COPD group (*P* < 0.05, Fig. 2A). Beta diversity significantly differed between the BE-FAO, BE, and COPD groups (R^2^ = 0.025, *P* = 0.001, ADONIS PERMANOVA). Notably, the COPD group differed considerably from the BE-FAO (R^2^ = 0.0203, adjusted *P* = 0.0015) and BE groups (R^2^ = 0.0219, adjusted *P* = 0.0015). However, the diversity indices were similar in the BE and BE-FAO groups, suggesting substantial overlaps in their microbiome profiles (R^2^ = 0.0107, adjusted *P* = 0.4370, detailed in Fig. 2B).

At the phylum level, patients with BE-FAO had higher *Proteobacteria* and lower *Firmicutes* levels than patients with COPD (Figure S3). No significant differences were found in four major phyla between the BE and BE-FAO groups. A detailed analysis of the ASVs annotated to species revealed that six taxa were significantly enriched in the COPD group, in contrast to the higher levels of *Pseudomonas aeruginosa* in the BE-FAO group (Figure S4). These microbial distributions were consistent with the conventional culture results detailed in Table 2. The concordance rate between the results of 16S rRNA gene sequencing and culture-based identification was 64.9% at the species level and 66.7% at the genus level (Table S2). Additionally, the detailed stacked plot in Figure S5 illustrates the relative abundance of the aforementioned taxa in the COPD, BE, and BE-FAO groups.

**Fig. 3:**
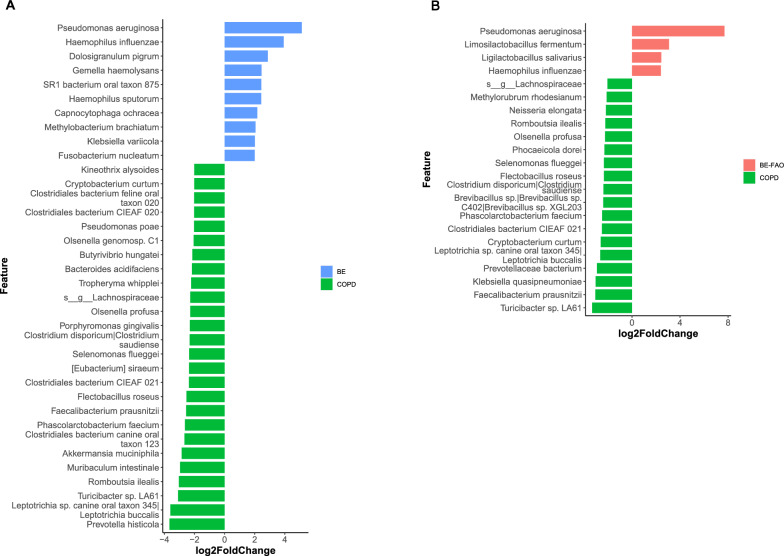
The differential abundance of lung microbiome analysis using DEseq2 in COPD, BE and BE-FAO groups (adjust gender and smoking status). The different taxonomic levels (adjusted *P* < 0.05 and fold change > 2.0) at species level in BE versus COPD groups (**A**) and in BE-FAO versus COPD groups (**B**). *BE* Bronchiectasis without fixed airflow obstruction, *BE-FAO* Bronchiectasis with fixed airflow obstruction, *COPD* Chronic obstructive pulmonary disease

After adjustment for gender and smoking status, our differential abundance analysis using DESeq2 indicated that the groups differed in their microbiome profiles (adjusted *P* < 0.05 and fold change > 2). At the species level, the BE group had enriched *Pseudomonas aeruginosa* and *Haemophilus influenzae*, in contrast to the high levels of commensal species in the COPD group (Fig. 3A). Moreover, unlike the COPD group, the BE-FAO group had a predominance of *Pseudomonas aeruginosa, Limosilactobacillus fermentum, Ligilactobacillus salivarius*, and *H. influenzae* (Fig. 3B).

### Bronchiectasis with FAO exhibits neutrophilic inflammation and specific microbiota compared to BE and COPD

**Fig. 4:**
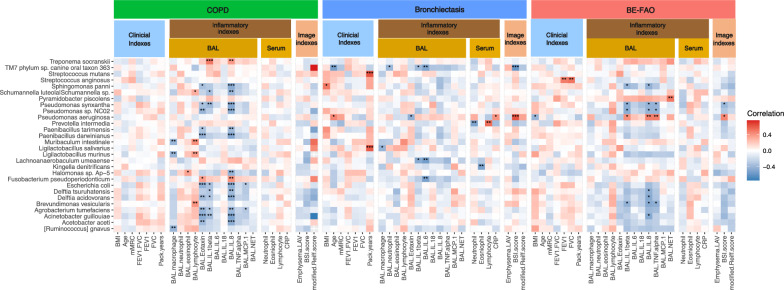
Heatmap showing spearman correlation between clinical variables and microbiome in COPD, BE and BE-FAO groups. Clinical variables are grouped into three categories: clinical indexes, inflammatory indexes, and imaging indexes. Only those taxa that displayed at least one significant correlation (q < .01, following FDR correction) were selected. The color-coded matrix represents the Spearman correlation coefficient, with red indicating a positive correlation and blue indicating a negative correlation. FDRs are denoted: *q < 0.05; **q < 0.01; ***q < 0.001. *BAL* Bronchoalveolar lavage, *BE* Bronchiectasis without fixed airflow obstruction, *BE-FAO* Bronchiectasis with fixed airflow obstruction, *BMI* Body Mass Index, *BSI* Bronchiectasis severity index, *CAT* COPD Assessment Test, *COPD* Chronic obstructive pulmonary disease, *CRP* C-reactive protein, *FDR* False discovery rate, *FEV*_*1*_ forced expiratory volume in 1 s, *FVC* forced vital capacity, *LAV* low-attenuation volume, *mMRC* modified Medical Research Council, *IL-1β* interleukin [IL]-1β, *IL-6* interleukin [IL]-6, *IL-8* interleukin [IL]-8, *IL-18* interleukin [IL]-18, *MCP-1* Monocyte chemoattractant protein-1, *NETs* Neutrophil extracellular traps, *TNF-α* tumor necrosis factor [TNF]-α

In comparison with patients having COPD alone, those with BE-FAO exhibited significantly elevated levels of BAL neutrophils and increased concentrations of neutrophilic inflammatory cytokines: Interleukin (IL)-1β, IL-6, IL-8, Monocyte Chemoattractant Protein-1 (MCP-1), and Tumor Necrosis Factor-alpha (TNF-α), as detailed in Table 2. Additionally, the BE-FAO group displayed higher levels of IL-8 and NETs compared to the BE group, despite presenting similar lung microbiome profiles. Further analysis, illustrated in Fig. 4, explores the correlations between clinical variables and specific lung bacterial taxa across the COPD, BE, and BE-FAO groups. Notably, *Pseudomonas aeruginosa* was positively correlated with airway neutrophilic cytokines and was associated with increased bronchiectasis severity (BSI score) and lower BMI in the BE-FAO group.

### Differences in clinical features, airway inflammation, and lung microbiome among patients with bronchiectasis with FAO according to ROSE criteria

**Fig. 5:**
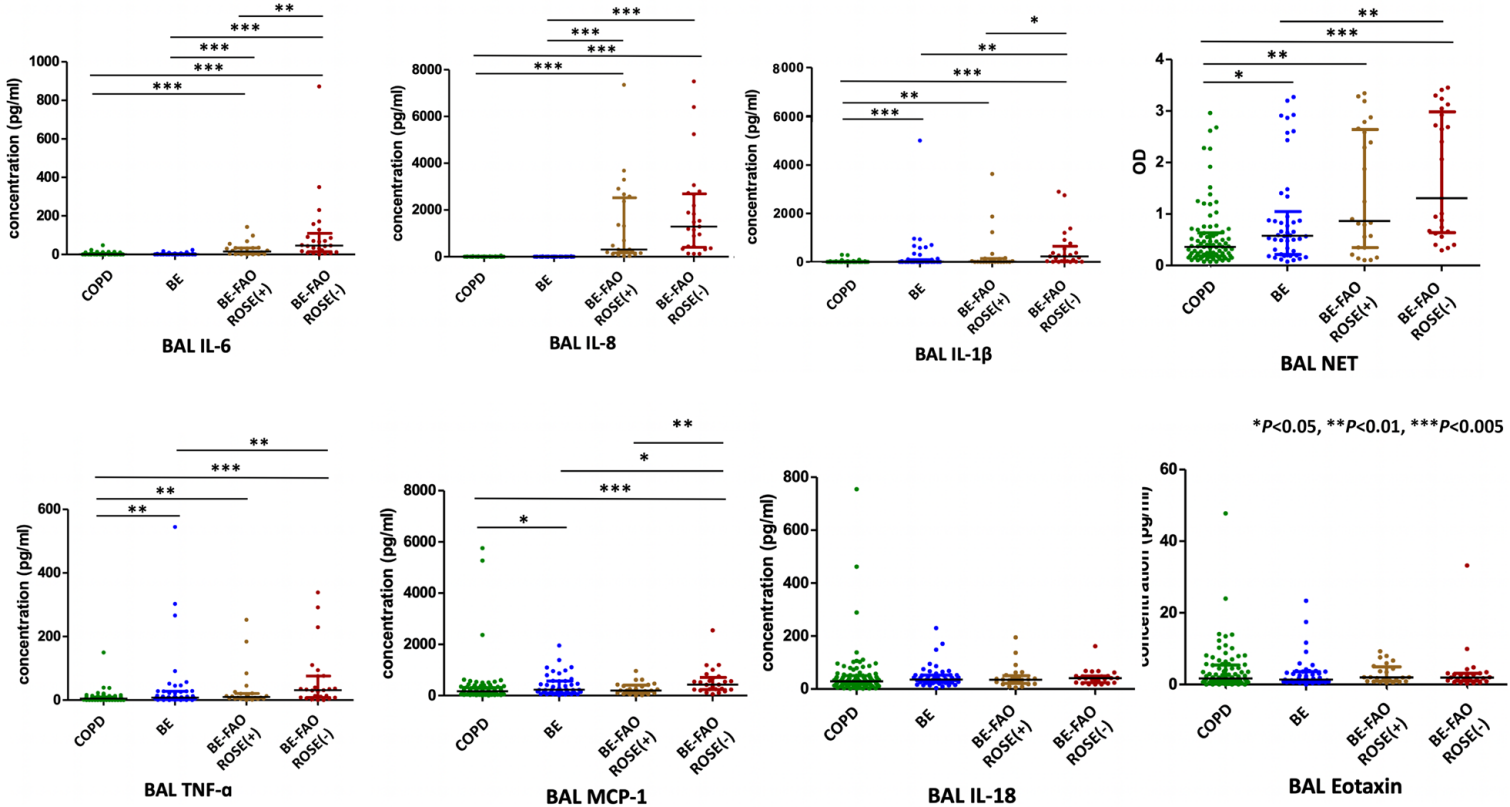
Differences in airway inflammatory profiles based on BAL samples in patients with COPD, BE, BE-FAO ROSE (+), and BE-FAO ROSE (−). The bronchoalveolar lavage (BAL) samples from study subjects were applied for multiplex Immunoassays. (*P < 0.05, **P < 0.01, ***P < 0.005). *BAL* bronchoalveolar lavage, *BE* Bronchiectasis without fixed airflow obstruction, *BE-FAO* Bronchiectasis with fixed airflow obstruction, *COPD* Chronic obstructive pulmonary disease, *IL-1* Interleukin [IL]-1β, *IL-6* Interleukin [IL]-6, *IL-18* Interleukin [IL]-18, *IL-8* Interleukin [IL]-8, *MCP-1* Monocyte chemoattractant protein-1, *NETs* neutrophil extracellular traps, *ROSE* Radiology, Obstruction, Symptoms, Exposure, *TNF-α* tumor necrosis factor [TNF]-α

We analyzed clinical variables and clinical outcomes in 49 bronchiectasis patients with FAO, distinguishing between those who met (n = 24) and did not meet (n = 25) the ROSE criteria, as detailed in Fig. 1 and Tables S3 and S4. Patients meeting the ROSE criteria, also known as the “COPD-bronchiectasis association,” were predominantly male, often smokers, and generally older. They exhibited a tendency towards COPD-related etiologies, presented with higher dyspnea and emphysema scores on HRCT scans, and showed elevated blood eosinophil and lymphocyte levels (Fig. 5).

By contrast, those not meeting the ROSE criteria, who formed a BE-FAO ROSE (−) group, were predominantly female and more likely to have idiopathic etiologies. These patients had significantly elevated levels of neutrophilic inflammatory cytokines, specifically IL-1β, IL-6, and MCP-1, in the BAL samples (Fig. 5). Despite these differences, no significant variation was found in lung function indices, bronchiectasis severity, usage of inhaled medications, bacterial culture results, and exacerbation rates between the groups (Tables S3 and S4).

**Fig. 6:**
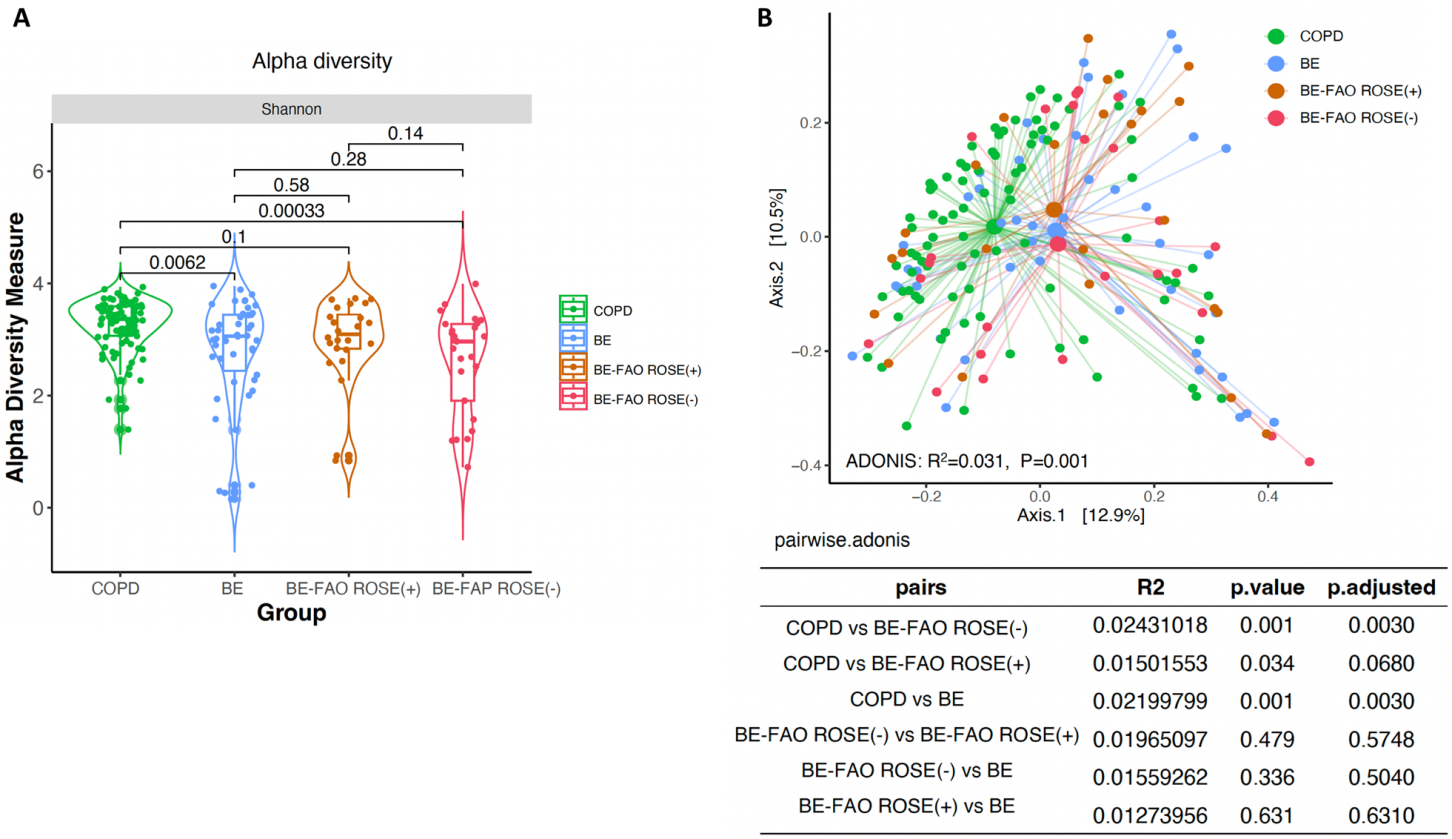
Alpha diversity (**A**) and beta diversity (**B**) of patients based on lung microbiome profiles. **A** BE, BE-FAO ROSE (+), and BE-FAO ROSE (−) patients showed comparable alpha diversity levels. **B** Marked differences emerged in beta diversity between the BE-FAO ROSE (−) and COPD groups (adjusted P = 0.003), while the differences between BE-FAO ROSE (+) and COPD were less pronounced (adjusted P value = 0.068). *BE* Bronchiectasis without fixed airflow obstruction, *BE-FAO* Bronchiectasis with fixed airflow obstruction, *COPD* Chronic obstructive pulmonary disease, *ROSE* Radiology, Obstruction, Symptoms, Exposure

In patients with BE-FAO, regardless of the ROSE status, similar alpha diversity and beta diversity were found for the lung microbiota communities, as depicted in Fig. 6A and B. Crucially, alpha diversity in the BE-FAO ROSE (−) group was significantly lower than that in the COPD group (*P* < 0.001). Alpha diversity was similar in the BE-FAO ROSE (+) group and the COPD group (*P* = 0.1). Furthermore, the pairwise analysis revealed marked differences in beta diversity between the COPD group and the BE-FAO ROSE (−) group (ADONIS PERMANOVA R^2^ = 0.024, adjusted *P* = 0.003). However, these differences were less pronounced between the COPD group and the BE-FAO ROSE (+) group (ADONIS PERMANOVA R^2^ = 0.015, *P* = 0.034, adjusted *P* = 0.068).

The BE-FAO ROSE (+) group had a notably higher relative abundance of *Candidatus Absconditabacteria* (*P* = 0.034). The BE-FAO ROSE (−) group exhibited a slightly increased, but not statistically significant, abundance of *Pseudomonas aeruginosa* (*P* = 0.086) (Figures S6 and S7). After adjustment for age and gender, DESeq2 analysis revealed that the BE-FAO ROSE (−) group had higher levels of species such as *Pseudoleptotrichia goodfellowii* and *Streptococcus mutans* than the ROSE (+) group (Figure S8).

### Association of specific lung bacterial taxa and airway inflammation with risk of future exacerbations in BE-FAO

**Fig. 7:**
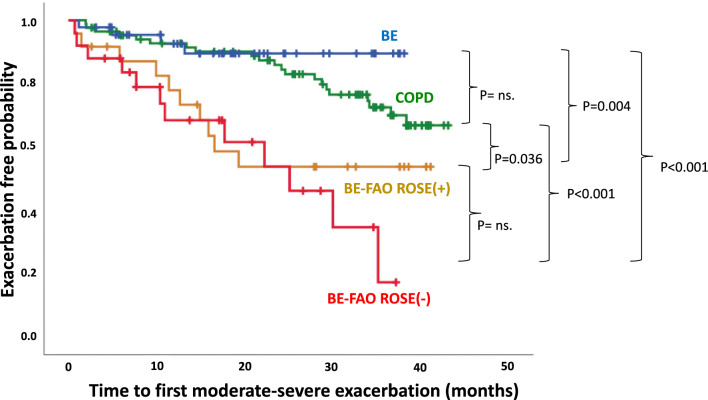
Time to first moderate-severe exacerbation: comparing COPD, BE, and BE-FAO (incorporating ROSE (+) and ROSE (−) subgroups). ns: not significant. *BE* Bronchiectasis without fixed airflow obstruction, *BE-FAO* Bronchiectasis with fixed airflow obstruction, *COPD* Chronic obstructive pulmonary disease, *ROSE* Radiology, Obstruction, Symptoms, Exposure

During a median follow-up of 2.46 years (range, 1.45–3.10), 47 participants (25.9% of those enrolled) experienced moderate-to-severe exacerbations, totaling 80 episodes. The BE-FAO group, including the ROSE (+) and ROSE (−) subgroups, had a significantly higher risk of exacerbations than the COPD and BE groups (Fig. 7). Clinically, as detailed in Table S5, patients with BE-FAO with a higher risk of exacerbations had higher blood neutrophil counts and levels of neutrophilic inflammatory cytokines (IL-1β and IL-8) in the BAL samples as well as lower FVC scores.

**Fig. 8:**
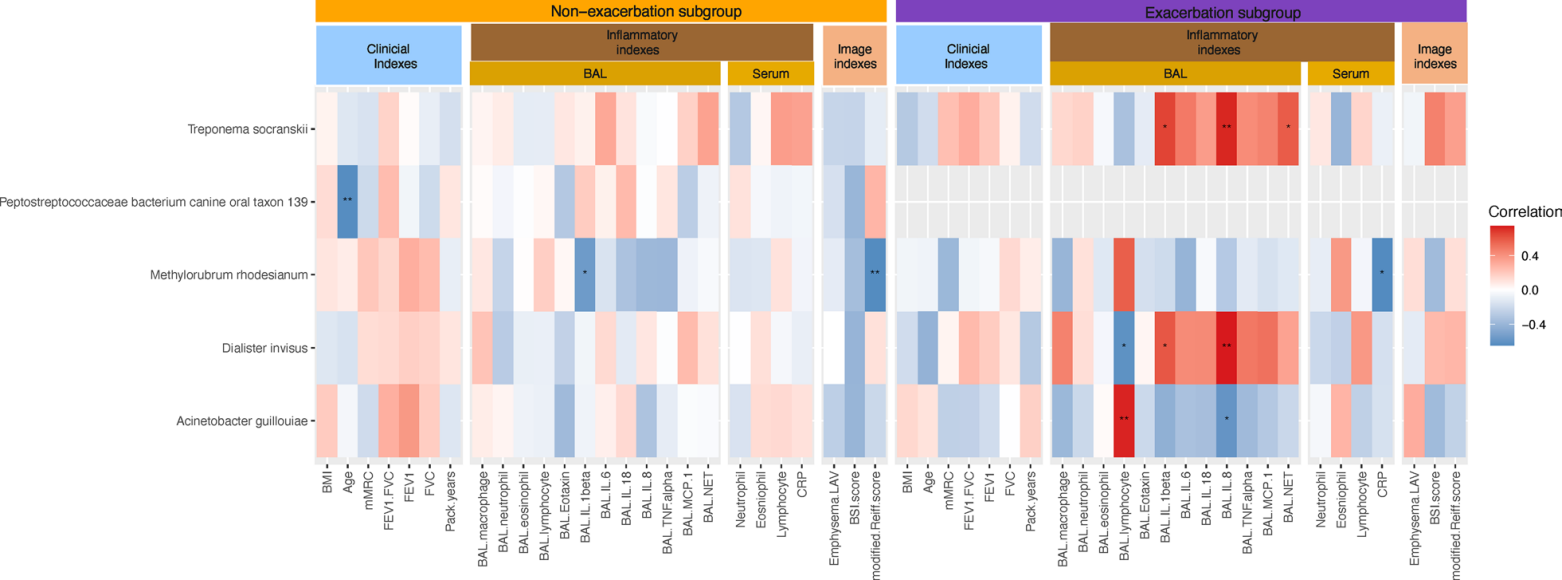
The correlation of clinical variables and lung microbiota in the bronchiectasis with FAO group. Heatmap showing spearman correlation between clinical variables and BAL microbiome in exacerbation subgroup and non-exacerbation subgroup. Clinical variables are grouped into three categories: clinical indexes, inflammatory indexes, and imaging indexes. Only those taxa that displayed at least one significant correlation (q < .01, following FDR correction) were selected. The color-coded matrix represents the Spearman correlation coefficient, with red indicating a positive correlation and blue indicating a negative correlation. FDRs are denoted: *q < 0.05; **q < 0.01; ***q < 0.001. The spearmans correlation revealed two oral taxa, *Treponema socranskii* and and *Dialister invisus*, in exacerbation group of BE-FAO were positively associated neutrophilic cytokines (BAL-IL 1β and BAL-IL 8)*.* *BAL* Bronchoalveolar lavage, *BE* Bronchiectasis without fixed airflow obstruction, *BE-FAO* Bronchiectasis with fixed airflow obstruction, *BMI* Body Mass Index, *BSI* Bronchiectasis severity index, *CAT* COPD Assessment Test, *COPD* Chronic obstructive pulmonary disease, *CRP* C-reactive protein, *FDR* False discovery rate, *FEV*_*1*_ forced expiratory volume in 1 s, *FVC* forced vital capacity, *LAV* low-attenuation volume, *mMRC* modified Medical Research Council, *IL-1β* interleukin [IL]-1β, *IL-6* interleukin [IL]-6, *IL-8* interleukin [IL]-8, *IL-18* interleukin [IL]-18, *MCP-1* Monocyte chemoattractant protein-1, *NETs* Neutrophil extracellular traps, *TNF-α* tumor necrosis factor [TNF]-α

Results from the lung microbiome analysis revealed similar alpha diversity (*P* = 0.12) and beta diversity (R^2^ = 0.025, *P* = 0.24) in the exacerbation and non-exacerbation subgroups of the BE-FAO group (Figure S9). Despite this similarity, the exacerbation subgroup tended to exhibit a higher relative abundance of Proteobacteria (*P* = 0.075), although the finding was nonsignificant (Figure S10). Further DESeq2 analysis identified a predominance of specific pathogens such as *Leptotrichia* sp. *canine oral taxon* 345, *Haemophilus parahaemolyticus*,* Pseudomonas aeruginosa, Bacteroides pyogenes*, and *Tropheryma whipplei* in the exacerbation subgroup relative to the non-exacerbation subgroup (Figure S11). Notably, two oral species—*Treponema socranskii* and *Dialister invisus*—were significantly correlated with higher levels of neutrophilic cytokines (BAL-IL 1β and BAL-IL 8), highlighting their potential role in the risk of exacerbations in the BE-FAO group (Fig. 8).

## Discussion

Our study represents a pioneering effort to analyze lung microbiota and airway inflammation in bronchiectasis patients with FAO from an East Asian population, comparing these patients with those having COPD and bronchiectasis without FAO using BAL samples. We discovered that the lung microbiota in patients with BE-FAO closely resembled that of patients with bronchiectasis, with both groups exhibiting reduced microbial diversity and a predominance of *Proteobacteria* compared to COPD patients alone. Bronchiectasis patients with FAO demonstrated greater neutrophilic airway inflammation and a higher risk of exacerbations than those with COPD or bronchiectasis alone. Importantly, we identified a positive correlation between *Pseudomonas aeruginosa* colonization and increased airway neutrophilic inflammation, along with a higher BSI score, potentially indicating a predictor for future exacerbations in the BE-FAO group. Furthermore, this is the first study to investigate two distinct entities within the BE-FAO group based on the ROSE (Radiology, Obstruction, Symptoms, and Exposure) criteria, revealing two unique endotypes characterized by their clinical characteristics, inflammatory patterns, and microbiome compositions.

Bronchiectasis and COPD often coexist, leading to the terms “COPD-bronchiectasis association” [16] or “Bronchiectasis-COPD overlap” [9, 15]. This overlap is associated with increased airway inflammation, more clinical symptoms, greater disease severity, and a worse prognosis than either disease alone [6, 9–11, 15]; these findings are consistent with our study. Differing from previous studies [13, 22], our research extended beyond just bronchiectasis patients meeting the ROSE criteria, commonly referred to as the “COPD-bronchiectasis association” [16]. We also included non-smoking advanced bronchiectasis patients in the BE-FAO group, which could be classified as “BE-FAO ROSE (−)”. Our results indicated that patients with BE-FAO, whether ROSE (+) or ROSE (−), and those with bronchiectasis alone, had comparable lung microbiomes. These findings are consistent with those of Huang et al. [22], who also employed the ROSE criteria. However, in contrast to the UK cohort [22], we observed that alpha diversity in BE-FAO ROSE (+) was similar to COPD, with less distinct beta diversity. The differences between the cohorts could be attributed to several factors: (1) The majority of our COPD and BE-FAO ROSE (+) cohort are males (> 95%), more likely to have smoking habits [24, 51] than the non-Asian cohort [22]. (2) Geographic variations in lung microbiome influenced by different COPD [24, 25, 51] and bronchiectasis [6, 26] risk factors and etiologies. (3) Environmental exposure, such as air or indoor pollution, along with geographic differences and varying dietary exposures, impacts the airway microbiome [6, 23, 25, 26]. (4) Ethnic-based differences in microbiome and host immunity interactions could also be a contributing factor [6, 23, 26].

Moreover, although the ROSE criteria effectively stratify patient groups, their primary reliance on smoking history may oversimplify the complexities of disease dynamics. These criteria do not account for other significant factors affecting disease development, such as genetic or environmental influences (e.g., exposure to indoor pollution) or pre-existing comorbidities. Thus, broader criteria should be established. Further research involving validation cohorts from diverse geographic regions is essential to expand these findings and provide a more comprehensive understanding of the multifactorial influences on diseases.

Furthermore, patients with the two disease entities of BE-FAO exhibited similar microbial diversity, with overlapping lung microbiota communities. Nevertheless, subtle differences emerged at the phyla and species levels. For instance, *Candidatus Absconditabacteria* was more common in BE-FAO ROSE (+), while species such as *Pseudoleptotrichia goodfellowii* and *Streptococcus mutans* were more prevalent in BE-FAO ROSE (−). Moreover, compared to patients with BE-FAO ROSE (+) or the “COPD-bronchiectasis association”, those with BE-FAO ROSE (−) were predominantly female and tended to have an idiopathic etiology, exhibited greater airway neutrophilic inflammatory cytokines, and had a lower emphysema score. However, the radiological severity of bronchiectasis, the degree of lung function obstruction, and exacerbation outcomes were similar between both entities. Given the variations in clinical features, etiologies, inflammatory profiles, and lung microbiome between these two entities of bronchiectasis with FAO, we hypothesize that they might represent distinct biological and microbiological endotypes. Further research incorporating more comprehensive microbial analyses, larger sample sizes, and broader cohorts is essential to validate these subtle differences and delve deeper into their potential impacts on disease dynamics and the clinical implications of their association.

Our analysis revealed a positive association between *Pseudomonas aeruginosa* colonization and neutrophilic inflammation, as well as higher severity of bronchiectasis in patients with FAO. This indicates a “*Proteobacteria*-neutrophilic endotypes” in the COPD–bronchiectasis association [22], potentially contributing to a higher risk of future exacerbations in BE-FAO and serving as a biomarker for poorer prognosis [15, 21, 52]. In contrast, the lung microbiome of patients with COPD exhibited greater diversity with a dominance of the *Firmicutes* phylum and commensal taxa, differing from those in BE and BE-FAO. This diversity suggests “diverse endotypes” in the COPD–bronchiectasis association [22], potentially associated with a lower risk of exacerbation compared to bronchiectasis with FAO. Prior research links high blood eosinophils with a *Firmicutes*-dominated microbiome [17, 19], supporting the effectiveness of inhaled corticosteroids (ICS) in COPD [19, 53]. The impact of eosinophils on the bronchiectasis microbiota is an area of growing interest [54, 55], with potential ICS benefits for specific bronchiectasis subgroups [55, 56]. Our East Asian cohort showed no clear correlation between blood eosinophils and specific lung microbiota in terms of clinical outcomes. Nevertheless, the directionality and causality of the relationship between airway inflammation and lung microbiome remain unclear. Longitudinal studies are crucial to ascertain whether changes in microbiota precede or follow changes in inflammation and to explore ongoing changes in the lung microbiome and inflammatory markers. Such studies are essential for deeper insights into their impacts on disease progression and treatment outcomes, including the underlying mechanisms. While our current study does not delve into therapeutic applications directly, it establishes a foundation for subsequent investigations that could significantly impact clinical practices and patient outcomes.

Another novel finding from our study is the positive association of two anaerobic oral taxa, *Treponema socranskii* and *Dialister invisus*, commonly detected in periodontitis [57, 58], with airway neutrophilic inflammation in the exacerbation subgroup of the BE-FAO group. This suggests that microaspiration-derived microbiota contribute to lung inflammation [59] and are associated with defective mucosal immunity in patients with chronic lung diseases [60–62]. Nevertheless, the specific role of the oral microbiome in patients with bronchiectasis and FAO, particularly its interaction with mucociliary clearance dysfunction, remains unexplored. Further studies are essential to investigate these relationships and their potential implications in the pathogenesis of these conditions.

Our study presents several limitations. First, as a prospective cross-sectional observational cohort study, our research primarily identifies associations rather than causality, emphasizing the necessity for longitudinal studies to clarify the causative links between the lung microbiome, airway inflammation, and clinical outcomes. Second, the study relies on 16S rRNA gene sequencing, which may not provide the necessary resolution to identify specific bacterial species or strains, nor does it yield functional information about the lung microbiome. Whole genome sequencing (WGS) is recommended to provide a more comprehensive understanding of the mechanistic pathways involved. Third, we enrolled only patients in clinically stable conditions to ensure patient safety for BAL collection and to minimize the impact of recent antibiotic exposure. Therefore, our conclusions might not be extrapolated to patients in the exacerbation phase or reflect the condition of those with more severe disease. Future studies should include broader patient populations, including patients with exacerbations and patients who were under antibiotic treatment, as well as healthy controls, to more comprehensively assess microbiome dynamics and its implications for different disease states. Fourth, although our study provides valuable insights, it serves as a discovery phase study primarily conducted in East Asian populations. Differences in microbiota composition across geographic regions and ethnic groups may limit the broad applicability of our results, underscoring the need for larger, multicenter trials to substantiate and generalize our findings across diverse geographical and ethnic contexts. Fifth, the sample size within each group may not adequately represent their respective populations. This could potentially limit the statistical power, especially when detecting minor effects or rare microbial species. Further study with a larger sample size is warranted to clarify these results. Lastly, our study focused on moderate-to-severe exacerbations in patients with COPD and bronchiectasis, yet it may have overlooked milder exacerbation events that often do not necessitate changes in medical treatment and are less likely to be documented in medical records. Future research may aim to capture and analyze the full spectrum of exacerbations to ensure a comprehensive understanding of their impacts.

## Conclusion

In the East Asian cohort, bronchiectasis with FAO is markedly distinct and clinically more severe compared to COPD or bronchiectasis alone, exhibiting increased neutrophilic inflammation and a higher risk of exacerbations. Both bronchiectasis with and without FAO, characterized by reduced microbial diversity and dominant *Proteobacteria*, share similar microbiome compositions, distinct from COPD alone. Utilizing the ROSE criteria, our study identified two distinct endotypes within the BE-FAO group, differentiated by their clinical features, inflammatory patterns, and microbiome attributes. Notably, a significant correlation was observed between *Pseudomonas aeruginosa* colonization and heightened airway neutrophilic inflammation in BE-FAO patients, along with an increased BSI score. This relationship may serve as an indicator of potential future exacerbations in the BE-FAO group.

### Supplementary Information

Supplementary Material 1. Figure S1. The alpha diversity (A) and beta diversity (B) of bronchoalveolar lavage (BAL), oral washing control (OWC) and negative control (NC) samples before decontam method. BAL samples (N=181, green dots), OWC samples (N=78, red dots) and NC samples including Bronchial washing control (BWC) (n=5, deep blue dots), Normal saline control (NSC) (n=5, light blue dots), Phosphate buffered saline (PBS) control (n=5, cyan blue dots), Extraction kit control (EKC) (n=8, deep purple dots), Non-Template control (NTC) (n=5, light purple dots).

Supplementary Material 2. Figure S2. The alpha diversity and beta diversity of bronchoalveolar lavage (BAL) (N=181) and oral washing control (OWC) (N=78) samples after removing the background contamination taxa. The microbiome analysis showed that BAL samples and OWC displayed significantly different. A, alpha‐diversity (*P*<0.001). B, Principal coordinates analysis (PCoA) showed significant separation microbial communities between the BAL and OWC samples (R^2^=0.293, *P-*value =0.001).

Supplementary Material 3. Figure S3. The distribution of relative abundance of top 10 major taxonomic groups in three groups at phylum level. The patients with BE-FAO had a higher relative abundance of *Proteobacteria* (p=0.011) and lower abundance of *Firmicutes* (p=0.0092) relative to the patients with COPD. No significant difference was observed in the proportions of the four major phyla in BE and BE-FAO. BE=Bronchiectasis without fixed airflow obstruction; BE-FAO= Bronchiectasis with fixed airflow obstruction; COPD=Chronic obstructive pulmonary disease.

Supplementary Material 4. Figure S4. Highlights species-level taxonomic distribution differences between COPD, BE, and BE-FAO patients. Among ASV annotated to specie, the COPD group showed higher prevalence of *Streptococcus parasanguinis*, *Schaalia odontolytica, Veillonella atypica, Lancefieldella parvula, Solobacterium moorei*, and *TM7 phylum sp canine oral taxon 250*, while *Pseudomonas aeruginosa* was more abundant in the BE-FAO group. Wilcoxon rank-sum test was used to compare the relative abundance of taxa. BE=Bronchiectasis without fixed airflow obstruction; BE-FAO= Bronchiectasis with fixed airflow obstruction; COPD=Chronic obstructive pulmonary disease.

Supplementary Material 5. Figure S5. Stacked plot of relative abundance of taxa at the species level in each sample within COPD (n=86), BE (n=46) and BE-FAO (n=49) group. BE=Bronchiectasis without fixed airflow obstruction; BE-FAO= Bronchiectasis with fixed airflow obstruction; COPD=Chronic obstructive pulmonary disease.

Supplementary Material 6. Figure S6. The difference of lung microbiota composition of patients in BE-FAO ROSE (+) (n=24) and BE-FAO ROSE (−) (n=25). The composition of major taxonomic groups and the distribution of relative abundance of phylum level. The patients with BE-FAO ROSE (+) had a higher relative abundance of *Candidatus Absconditabacteria* (*P*=0.034) at the phyla level compared to those with BE-FAO ROSE (−). BE-FAO= Bronchiectasis with fixed airflow obstruction; ROSE=Radiology, Obstruction, Symptoms, Exposure.

Supplementary Material 7. Figure S7. The difference of lung microbiota composition of patients in BE-FAO ROSE (+) (n=24) and BE-FAO ROSE (−) (n=25). The composition of major taxonomic groups and the distribution of relative abundance of species level. The patients with BE-FAO ROSE (−) had a relative abundance of *Pseudomonas* *aeruginosa* (*P*=0.086) when ASV annotated to species level, compared with those BE-FAO ROSE (+). BE-FAO= Bronchiectasis with fixed airflow obstruction; ROSE=Radiology, Obstruction, Symptoms, Exposure.

Supplementary Material 8. Figure S8. The differential abundance of lung microbiome analysis using DEseq2 (after adjusting for age and gender) in the BE-FAO group. The different taxonomic levels (adjusted p<0.05 and fold change>2.0) in BE-FAO ROSE (+) versus BE-FAO ROSE (−) at (A) genus level (B) species level. We further disclosed that *Pseudoleptotrichia goodfellowii*, *Streptococcus mutans, Veillonella sp.oral taxon 780, Prevotella denticola, Capnocytophaga endodontalis, Loriellopsis cavernicola, Olsenella genomosp.C1 and Selenomonas sp. oral taxon *were enriched in BE-FAO ROSE (−) group compared to BE-FAO ROSE (+) group. BE-FAO= Bronchiectasis with fixed airflow obstruction; ROSE=Radiology, Obstruction, Symptoms, Exposure.

Supplementary Material 9. Figure S9 shows alpha (A) and beta (B) diversity in BE-FAO patients with future exacerbations (n=22) versus those without (n=27) using BAL microbiome profiles. Both alpha diversity (*P* = 0.12) and beta diversity (R^2^ = 0.025, *P* = 0.24) measures were similar between exacerbation and non-exacerbation subgroups. BE-FAO= Bronchiectasis with fixed airflow obstruction.

Supplementary Material 10. Figure S10. Differences in lung microbiota composition at the phylum level between patients with exacerbations (n = 22) and non-exacerbations (n = 27) in the BE-FAO group. In this group, the exacerbation subgroup had a higher relative abundance of *Proteobacteria* (*P *=0.075) compared with the non-exacerbation subgroup, although this difference was nonsignificant. No significant differences were obtained in other major phyla between the exacerbation and non-exacerbation subgroups. BE-FAO = bronchiectasis with FAO.

Supplementary Material 11. Figure S11. The differential abundance of lung microbiome analysis using DEseq2 in the BE-FAO group. The different taxonomic levels (adjusted p<0.05 and fold change>2.0) in exacerbation versus non-exacerbation subgroups at (A) phylum level (B) species level. DESeq2 analysis revealed that the exacerbation subgroup of BE-FAO had a predominance of *Leptotrichia sp. canine oral taxon 345, Haemophilus parahaemolyticus, Pseudomonas aeruginosa, Bacteroides pyogenes,* and *Tropheryma whipplei* relative to the non-exacerbation subgroup. BE-FAO= Bronchiectasis with fixed airflow obstruction.

Supplementary Material 12.

Supplementary Material 13.

Supplementary Material 14.

Supplementary Material 15.

Supplementary Material 16.

## Data Availability

The 16S rRNA gene sequence data used in this study are available from National Center for Biotechnology Information (NCBI) Sequence Read Archive (SRA) database under PRJNA924101： https://dataview.ncbi.nlm.nih.gov/object/PRJNA924101?reviewer = 6aivli35eho5jdrfoatvvpbf10.

## Abbreviations

BAL: Bronchoalveolar lavage
BE: Bronchiectasis
BE-FAO: Bronchiectasis with fixed airflow obstruction
BMI: Body Mass Index
BSI: Bronchiectasis severity index
CAT: COPD Assessment Test
COPD: Chronic obstructive pulmonary disease
FAO: Fixed airflow obstruction
FEV1: Forced expiratory volume in 1 s
FVC: Forced vital capacity
HU: Hounsfield unit
IL-1β: Interleukin [IL]-1β
IL-6: Interleukin [IL]-6
IL-8: Interleukin [IL]-8
IL-18: Interleukin [IL]-18
ICS: Inhaled corticosteroid
LABA: Long-acting β2 Sympathomimetic Agonists
LAMA: Long-acting muscarinic antagonist
LAV: Low-attenuation volume
MCP-1: Monocyte chemoattractant protein-1
mMRC: Modified Medical Research Council
NETs: Neutrophil extracellular traps
NTM: Non-tuberculosis mycobacteria
ROSE: Radiology, Obstruction, Symptoms, Exposure
TB: Tuberculosis
TNF-α: Tumor necrosis factor [TNF]-α
Vit D3: Vitamin D3

## References

[1] Flume PA, Chalmers JD, Olivier KN. Advances in bronchiectasis: endotyping, genetics, microbiome, and disease heterogeneity. Lancet (London, England). 2018;392:880–90. 30215383 10.1016/S0140-6736(18)31767-7 PMC6173801

[2] Polverino E, Goeminne PC, McDonnell MJ, Aliberti S, Marshall SE, et al. European Respiratory Society guidelines for the management of adult bronchiectasis. Eur Respir J. 2017;50:1700629. 28889110 10.1183/13993003.00629-2017

[3] Naidich DP, McCauley DI, Khouri NF, Stitik FP, Siegelman SS. Computed tomography of bronchiectasis. J Comput Assist Tomogr. 1982;6:437. 7096687 10.1097/00004728-198206000-00001

[4] Chalmers JD, Goeminne P, Aliberti S, McDonnell MJ, Lonni S, Davidson J, et al. The bronchiectasis severity index. An international derivation and validation study. Am J Respir Crit Care Med. 2014;189:576. 24328736 10.1164/rccm.201309-1575OC PMC3977711

[5] Goeminne PC, Nawrot TS, Ruttens D, Seys S, Dupont LJ. Mortality in non-cystic fibrosis bronchiectasis: a prospective cohort analysis. Respir Med. 2014;108:287. 24445062 10.1016/j.rmed.2013.12.015

[6] Dhar R, Singh S, Talwar D, Murali Mohan BV, Tripathi SK, Swarnakar R, et al. Clinical outcomes of bronchiectasis in India: data from the EMBARC/Respiratory Research Network of India registry. Eur Respir J. 2023;61:00611. 10.1183/13993003.00611-2022 PMC9816417 36229049

[7] Global Initiative for Chronic Obstructive Lung Disease. Global strategy for the diagnosis, management and prevention of COPD. 2022. Global initiative for chronic obstructive lung disease website. http://goldcopd.org/

[8] Polverino E, Dimakou K, Hurst J, Martinez-Garcia MA, Miravitlles M, Paggiaro P, et al. The overlap between bronchiectasis and chronic airway diseases: state of the art and future directions. Eur Respir J. 2018;52:00328. 10.1183/13993003.00328-2018 30049739

[9] Hurst JR, Elborn JS, De Soyza A, BRONCH-UK Consortium. COPD-bronchiectasis overlap syndrome. Eur Respir J. 2015;45:310. 25653262 10.1183/09031936.00170014

[10] Martínez-García MA, de la Rosa CD, Soler-Cataluña JJ, Donat-Sanz Y, Serra PC, Lerma MA, et al. Prognostic value of bronchiectasis in patients with moderate-to-severe chronic obstructive pulmonary disease. Am J Respir Crit Care Med. 2013;187:823. 23392438 10.1164/rccm.201208-1518OC

[11] Gatheral T, Kumar N, Sansom B, Lai D, Nair A, Vlahos I, et al. COPD-related bronchiectasis; independent impact on disease course and outcomes. COPD. 2014;11:605. 24983298 10.3109/15412555.2014.922174

[12] Du Q, Jin J, Liu X, Sun Y. Bronchiectasis as a comorbidity of chronic obstructive pulmonary disease: a systematic review and meta-analysis. PLoS ONE. 2016;11: e0150532. 26978269 10.1371/journal.pone.0150532 PMC4792534

[13] Tiew PY, Lim AYH, Keir HR, Dicker AJ, Mac Aogáin M, Pang SL, et al. High frequency of allergic bronchopulmonary aspergillosis in bronchiectasis-COPD overlap. Chest. 2022;161:40. 34364870 10.1016/j.chest.2021.07.2165

[14] Chalmers JD, Moffitt KL, Suarez-Cuartin G, Sibila O, Finch S, Furrie E, et al. Neutrophil elastase activity is associated with exacerbations and lung function decline in bronchiectasis. Am J Respir Crit Care Med. 2017;195:1384. 27911604 10.1164/rccm.201605-1027OC PMC5443898

[15] Tiew PY, Jaggi TK, Chan LLY, Chotirmall SH. The airway microbiome in COPD, bronchiectasis and bronchiectasis-COPD overlap. Clin Respir J. 2021;15:123. 33063421 10.1111/crj.13294

[16] Traversi L, Miravitlles M, Martinez-Garcia MA, Shteinberg M, Bossios A, Dimakou K, et al. ROSE: radiology, obstruction, symptoms and exposure—a Delphi consensus definition of the association of COPD and bronchiectasis by the EMBARC Airways Working Group. ERJ Open Res. 2021;7:00399. 34820447 10.1183/23120541.00399-2021 PMC8607072

[17] Dicker AJ, Huang JTJ, Lonergan M, Keir HR, Fong CJ, Tan B, et al. The sputum microbiome, airway inflammation, and mortality in chronic obstructive pulmonary disease. J Allergy Clin Immunol. 2021;147:158. 32353489 10.1016/j.jaci.2020.02.040

[18] Wang Z, Bafadhel M, Haldar K, Spivak A, Mayhew D, Miller BE, et al. Lung microbiome dynamics in COPD exacerbations. Eur Respir J. 2016;47:1082. 26917613 10.1183/13993003.01406-2015

[19] Wang Z, Locantore N, Haldar K, Ramsheh MY, Beech AS, et al. Inflammatory endotype-associated airway microbiome in chronic obstructive pulmonary disease clinical stability and exacerbations: a multicohort longitudinal analysis. Am J Respir Crit Care Med. 2021;203(12):1488–502. 33332995 10.1164/rccm.202009-3448OC PMC8483235

[20] Richardson H, Dicker AJ, Barclay H, Chalmers JD. The microbiome in bronchiectasis. Eur Respir Rev. 2019;28:0048. 10.1183/16000617.0048-2019 PMC9489022 31484665

[21] Araújo D, Shteinberg M, Aliberti S, Goeminne PC, Hill AT, Fardon TC, et al. The independent contribution of Pseudomonas aeruginosa infection to long-term clinical outcomes in bronchiectasis. Eur Respir J. 2018;51:01953. 10.1183/13993003.01953-2017 29386336

[22] Huang JT, Cant E, Keir HR, Barton AK, Kuzmanova E, Shuttleworth M, et al. Endotyping chronic obstructive pulmonary disease, bronchiectasis, and the “chronic obstructive pulmonary disease-bronchiectasis association.” Am J Respir Crit Care Med. 2022;206:417. 35436182 10.1164/rccm.202108-1943OC

[23] Yang IA, Jenkins CR, Salvi SS. Chronic obstructive pulmonary disease in never-smokers: risk factors, pathogenesis, and implications for prevention and treatment. Lancet Respir Med. 2022;10:497. 35427530 10.1016/S2213-2600(21)00506-3

[24] Hsieh MJ, Huang SY, Yang TM, Tao CW, Cheng SL, Lee CH, et al. The impact of 2011 and 2017 Global Initiative for Chronic Obstructive Pulmonary Disease (GOLD) guidelines on allocation and pharmacological management of patients with COPD in Taiwan: Taiwan Obstructive Lung Disease (TOLD) study. Int J Chron Obstruct Pulmon Dis. 2018;13:2949. 30310271 10.2147/COPD.S176065 PMC6165725

[25] Lin L, Yi X, Liu H, Meng R, Li S, Liu X, et al. The airway microbiome mediates the interaction between environmental exposure and respiratory health in humans. Nat Med. 2023;29:1750. 37349537 10.1038/s41591-023-02424-2

[26] Chandrasekaran R, Mac Aogáin M, Chalmers JD, Elborn SJ, Chotirmall SH. Geographic variation in the aetiology, epidemiology and microbiology of bronchiectasis. BMC Pulm Med. 2018;18:83. 29788932 10.1186/s12890-018-0638-0 PMC5964678

[27] Hill AT, Sullivan AL, Chalmers JD, De Soyza A, Elborn JS, Floto RA, et al. British Thoracic Society Guideline for bronchiectasis in adults. BMJ Open Respir Res. 2018;5: e000348. 30687502 10.1136/bmjresp-2018-000348 PMC6326298

[28] Hill AT, Haworth CS, Aliberti S, Barker A, Blasi F, Boersma W, et al. Pulmonary exacerbation in adults with bronchiectasis: a consensus definition for clinical research. Eur Respir J. 2017;49:1700051. 28596426 10.1183/13993003.00051-2017

[29] Morris A, Beck JM, Schloss PD, Campbell TB, Crothers K, Curtis JL, et al. Comparison of the respiratory microbiome in healthy nonsmokers and smokers. Am J Respir Crit Care Med. 2013;187:1067. 23491408 10.1164/rccm.201210-1913OC PMC3734620

[30] Amatullah H, Shan Y, Beauchamp BL, Gali PL, Gupta S, Maron-Gutierrez T, et al. DJ-1/PARK7 Impairs bacterial clearance in sepsis. Am J Respir Crit Care Med. 2017;195:889. 27735193 10.1164/rccm.201604-0730OC

[31] Gauthier TW, Grunwell JR, Ping XD, Harris FL, Brown LA. Impaired defenses of neonatal mouse alveolar macrophage with cftr deletion are modulated by glutathione and TGF-β1. Physiol Rep. 2017;5: e13086. 28325787 10.14814/phy2.13086 PMC5371544

[32] Lood C, Blanco LP, Purmalek MM, et al. Neutrophil extracellular traps enriched in oxidized mitochondrial DNA are interferogenic and contribute to lupus-like disease. Nat Med. 2016;22:146. 26779811 10.1038/nm.4027 PMC4742415

[33] Available from: https://www.qiagen.com/tw/products/discovery-and-translational-research/dna-rna-purification/dna-purification/microbial-dna/qiaamp-dna-microbiome-kit/?clear=true#orderinginformation

[34] Lin YC, Chen YT, Li KY, Carmona-Rivera C, De Ravin SS, Smith CK, et al. Investigating the mechanistic differences of obesity-inducing *Lactobacillus**kefiranofaciens* M1 and anti-obesity *Lactobacillus**mali* APS1 by microbolomics and metabolomics. Front Microbiol. 2020;11:1454. 32733406 10.3389/fmicb.2020.01454 PMC7360855

[35] Bolyen E, Rideout JR, Dillon MR, Bokulich NA, Abnet CC, Al-Ghalith GA, et al. Reproducible, interactive, scalable and extensible microbiome data science using QIIME 2. Nat Biotechnol. 2019;37:852. 31341288 10.1038/s41587-019-0209-9 PMC7015180

[36] Callahan BJ, McMurdie PJ, Rosen MJ, Han AW, Johnson AJ, Holmes SP. DADA2: high-resolution sample inference from Illumina amplicon data. Nat Methods. 2016;13:581. 27214047 10.1038/nmeth.3869 PMC4927377

[37] Camacho C, Coulouris G, Avagyan V, Ma N, Papadopoulos J, Bealer K, et al. BLAST+: architecture and applications. BMC Bioinform. 2009;10:421. 10.1186/1471-2105-10-421 PMC2803857 20003500

[38] McMurdie PJ, Holmes S. Phyloseq: an R package for reproducible interactive analysis and graphics of microbiome census data. PLoS ONE. 2013;8: e61217. 23630581 10.1371/journal.pone.0061217 PMC3632530

[39] Oksanen J, Blanchet FG, Friendly M, Kindt R, Legendre P, McGlinn D, et al. Community ecology package. R Package Ver. 2013;2:321.

[40] Barnett DJ, Arts IC, Penders J. microviz: an R package for microbiome data visualization and statistics. J Open Source Softw. 2021;6:3201. 10.21105/joss.03201

[41] Wickham H. Ggplot2: elegant graphics for data analysis. New York: Springer; 2016.

[42] Love MI, Huber W, Anders S. Moderated estimation of fold change and dispersion for RNA-seq data with DESeq2. Genome Biol. 2014;5:550. 10.1186/s13059-014-0550-8 PMC4302049 25516281

[43] Davis NM, Proctor DM, Holmes SP, Relman DA, Callahan BJ. Simple statistical identification and removal of contaminant sequences in marker-gene and metagenomics data. Microbiome. 2018;6:226. 30558668 10.1186/s40168-018-0605-2 PMC6298009

[44] Carney SM, Clemente JC, Cox MJ, Dickson RP, Huang YJ, Kitsios GD, et al. Methods in lung microbiome research. Am J Respir Cell Mol Biol. 2020;62:283. 31661299 10.1165/rcmb.2019-0273TR PMC7055701

[45] Dickson RP, Erb-Downward JR, Freeman CM, McCloskey L, Falkowski NR, Huffnagle GB, et al. Bacterial topography of the healthy human lower respiratory tract. MBio. 2017;8: e02287. 28196961 10.1128/mBio.02287-16 PMC5312084

[46] Dickson RP, Martinez FJ, Huffnagle GB. The role of the microbiome in exacerbations of chronic lung diseases. Lancet. 2014;384:691. 25152271 10.1016/S0140-6736(14)61136-3 PMC4166502

[47] Dickson RP, Cox MJ, et al. Sampling. In: Cox MJ, Ege MJ, et al., editors. The lung microbiome (ERS Monograph). Sheffield: European Respiratory Society; 2019. p. 1–17.

[48] Wang Z, Gu S, Leader JK, Kundu S, Tedrow JR, Sciurba FC, et al. Optimal threshold in CT quantification of emphysema. Eur Radiol. 2013;23:975. 23111815 10.1007/s00330-012-2683-z PMC3573224

[49] Lor KL, Liu CP, Chang YC, Yu CJ, Wang CY, Chung MJ, et al. Predictive modelling of lung function using emphysematous density distribution. Sci Rep. 2019;9:19763. 31875053 10.1038/s41598-019-56351-9 PMC6930211

[50] Reiff DB, Wells AU, Carr DH, Cole PJ, Hansell DM. CT findings in bronchiectasis: limited value in distinguishing between idiopathic and specific types. AJR Am J Roentgenol. 1995;165:261. 7618537 10.2214/ajr.165.2.7618537

[51] Liu X, Sun W, Ma W, Wang H, Xu K, Zhao L, et al. Smoking related environmental microbes affecting the pulmonary microbiome in Chinese population. Sci Total Environ. 2022;829: 154652. 35307427 10.1016/j.scitotenv.2022.154652

[52] Eklöf J, Sørensen R, Ingebrigtsen TS, Sivapalan P, Achir I, Boel JB, et al. Pseudomonas aeruginosa and risk of death and exacerbations in patients with chronic obstructive pulmonary disease: an observational cohort study of 22 053 patients. Clin Microbiol Infect. 2020;26:227. 31238116 10.1016/j.cmi.2019.06.011

[53] Martinez-Garcia MA, Faner R, Oscullo G, de la Rosa D, Soler-Cataluña JJ, Ballester M, et al. Inhaled steroids, circulating eosinophils, chronic airway infection, and pneumonia risk in chronic obstructive pulmonary disease. A network analysis. Am J Respir Crit Care Med. 2020;201:1078. 31922913 10.1164/rccm.201908-1550OC

[54] Shoemark A, Shteinberg M, De Soyza A, Haworth CS, Richardson H, Gao Y, et al. Characterization of eosinophilic bronchiectasis: a European multicohort study. Am J Respir Crit Care Med. 2022;205:894. 35050830 10.1164/rccm.202108-1889OC

[55] Singh D, Brightling C. Bronchiectasis, the latest eosinophilic airway disease: what about the microbiome? Am J Respir Crit Care Med. 2022;205:860. 35213295 10.1164/rccm.202201-0105ED PMC9838629

[56] Aliberti S, Sotgiu G, Blasi F, Saderi L, Posadas T, Martinez Garcia MA. Blood eosinophils predict inhaled fluticasone response in bronchiectasis. Eur Respir J. 2020;56:2000453. 32398295 10.1183/13993003.00453-2020

[57] Takeuchi Y, Umeda M, Sakamoto M, Benno Y, Huang Y, Ishikawa I. *Treponema**socranskii*, *Treponema**denticola*, and *Porphyromonas**gingivalis* are associated with severity of periodontal tissue destruction. J Periodontol. 2001;72:1354–63. 11699477 10.1902/jop.2001.72.10.1354

[58] Rôças IN, Siqueira JF Jr. Characterization of Dialister species in infected root canals. J Endod. 2006;32:1057. 17055906 10.1016/j.joen.2006.04.010

[59] Segal LN, Clemente JC, Tsay JC, Koralov SB, Keller BC, Wu BG, et al. Enrichment of the lung microbiome with oral taxa is associated with lung inflammation of a Th17 phenotype. Nat Microbiol. 2016;1:16031. 27572644 10.1038/nmicrobiol.2016.31 PMC5010013

[60] Mammen MJ, Scannapieco FA, Sethi S. Oral-lung microbiome interactions in lung diseases. Periodontol. 2000;2020(83):234. 10.1111/prd.12301 32385873

[61] Pathak JL, Yan Y, Zhang Q, Wang L, Ge L. The role of oral microbiome in respiratory health and diseases. Respir Med. 2021;185: 106475. 34049183 10.1016/j.rmed.2021.106475

[62] Dong J, Li W, Wang Q, Chen J, Zu Y, Zhou X, et al. Relationships between oral microecosystem and respiratory diseases. Front Mol Biosci. 2022;8: 718222. 35071321 10.3389/fmolb.2021.718222 PMC8767498

