# Supplementary figures and images for: The clinical impacts of lung microbiome in bronchiectasis with fixed airflow obstruction: a prospective cohort study
Source: Respir Res. 2024 Aug 14;25:308. doi: 10.1186/s12931-024-02931-x (PMC11325704; doi:10.1186/s12931-024-02931-x)

**A****Alpha diversity**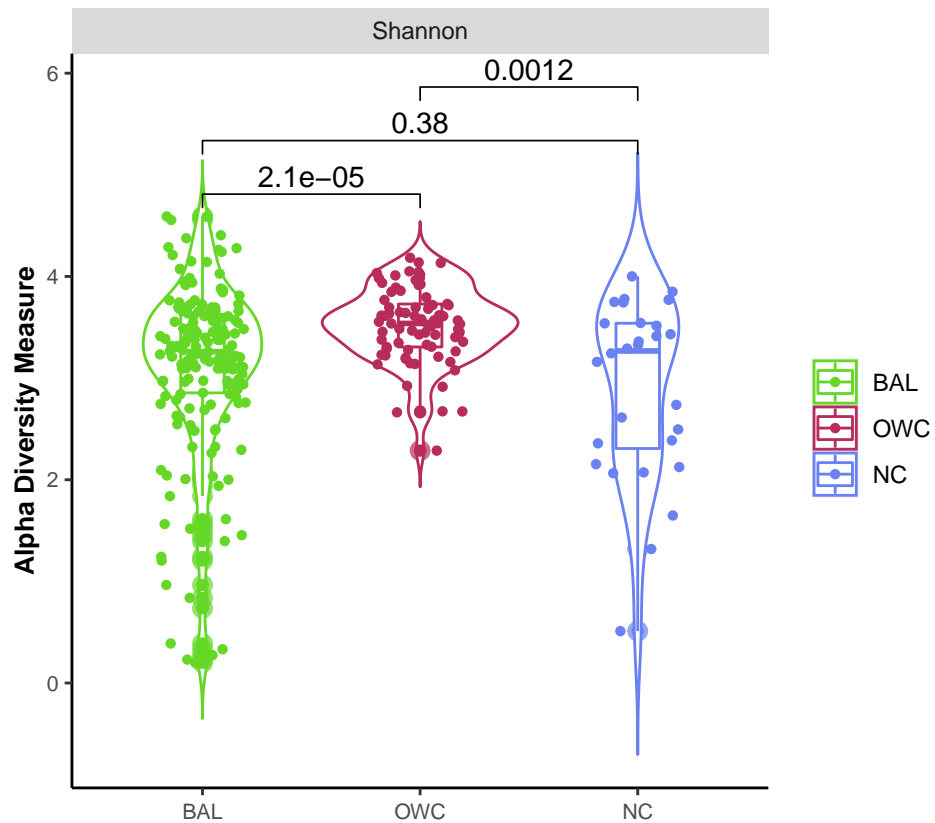**B****Beta diversity**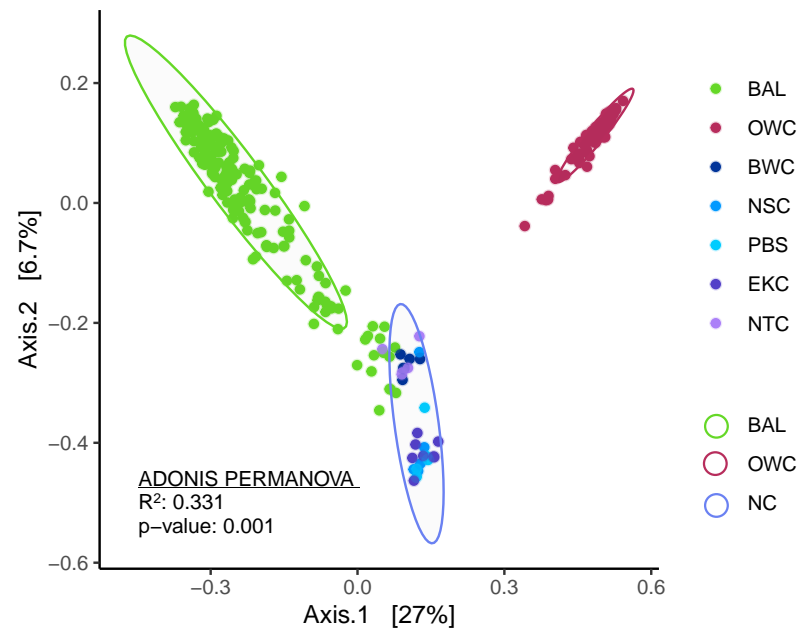

| pairs      | R <sup>2</sup> | p.value | adjusted.p |
|------------|----------------|---------|------------|
| BAL vs OWC | 0.2919421      | 0.001   | 0.001      |
| BAL vs NC  | 0.1023580      | 0.001   | 0.001      |
| OWC vs NC  | 0.2407252      | 0.001   | 0.001      |

Supplement: Supplementary file 1 — Supplementary Material 1. Figure S1. The alpha diversity (A) and beta diversity (B) of bronchoalveolar lavage (BAL), oral washing control (OWC) and negative control (NC) samples before decontam method. BAL samples (N=181, green dots), OWC samples (N=78, red dots) and NC samples including Bronchial washing control (BWC) (n=5, deep blue dots), Normal saline control (NSC) (n=5, light blue dots), Phosphate buffered saline (PBS) control (n=5, cyan blue dots), Extraction kit control (EKC) (n=8, deep purple dots), Non-Template control (NTC) (n=5, light purple dots). [file 12931_2024_2931_MOESM1_ESM.pdf]

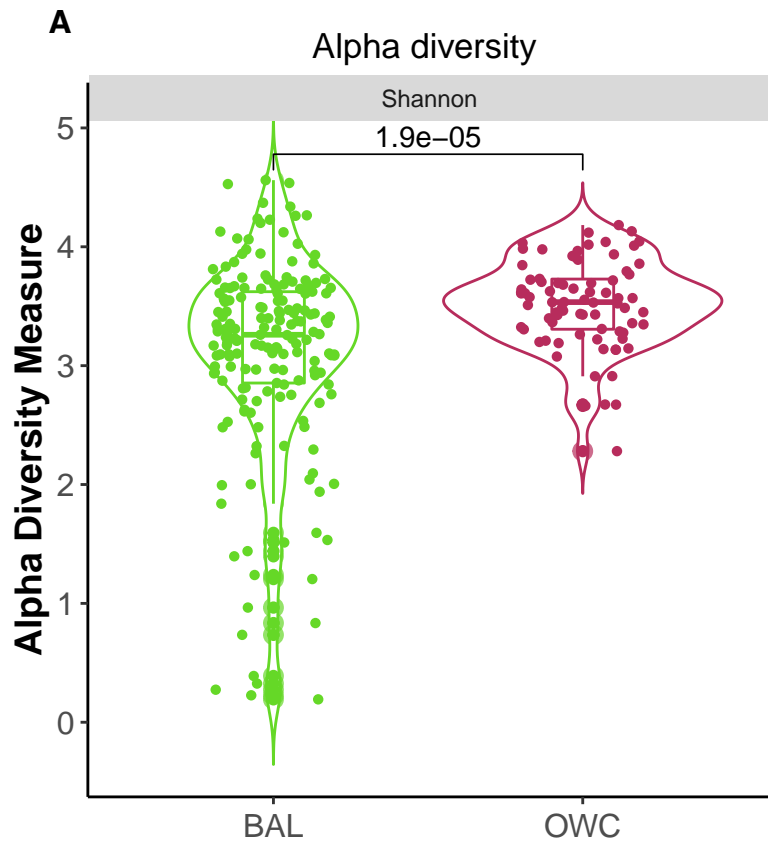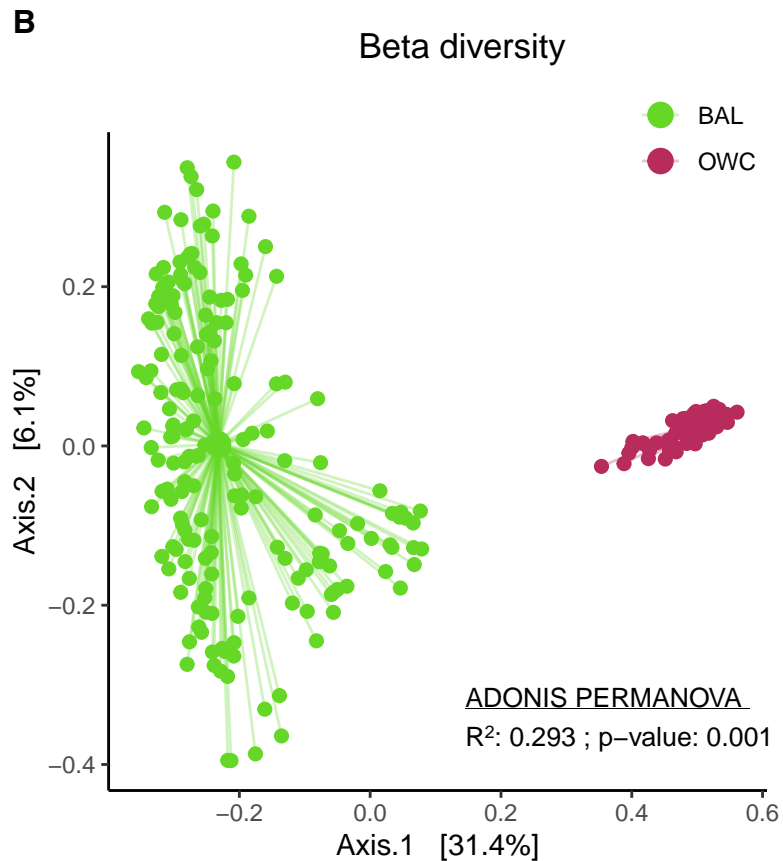

Supplement: Supplementary file 2 — Supplementary Material 2. Figure S2. The alpha diversity and beta diversity of bronchoalveolar lavage (BAL) (N=181) and oral washing control (OWC) (N=78) samples after removing the background contamination taxa. The microbiome analysis showed that BAL samples and OWC displayed significantly different. A, alpha‐diversity (P<0.001). B, Principal coordinates analysis (PCoA) showed significant separation microbial communities between the BAL and OWC samples (R2=0.293, P-value =0.001). [file 12931_2024_2931_MOESM2_ESM.pdf]

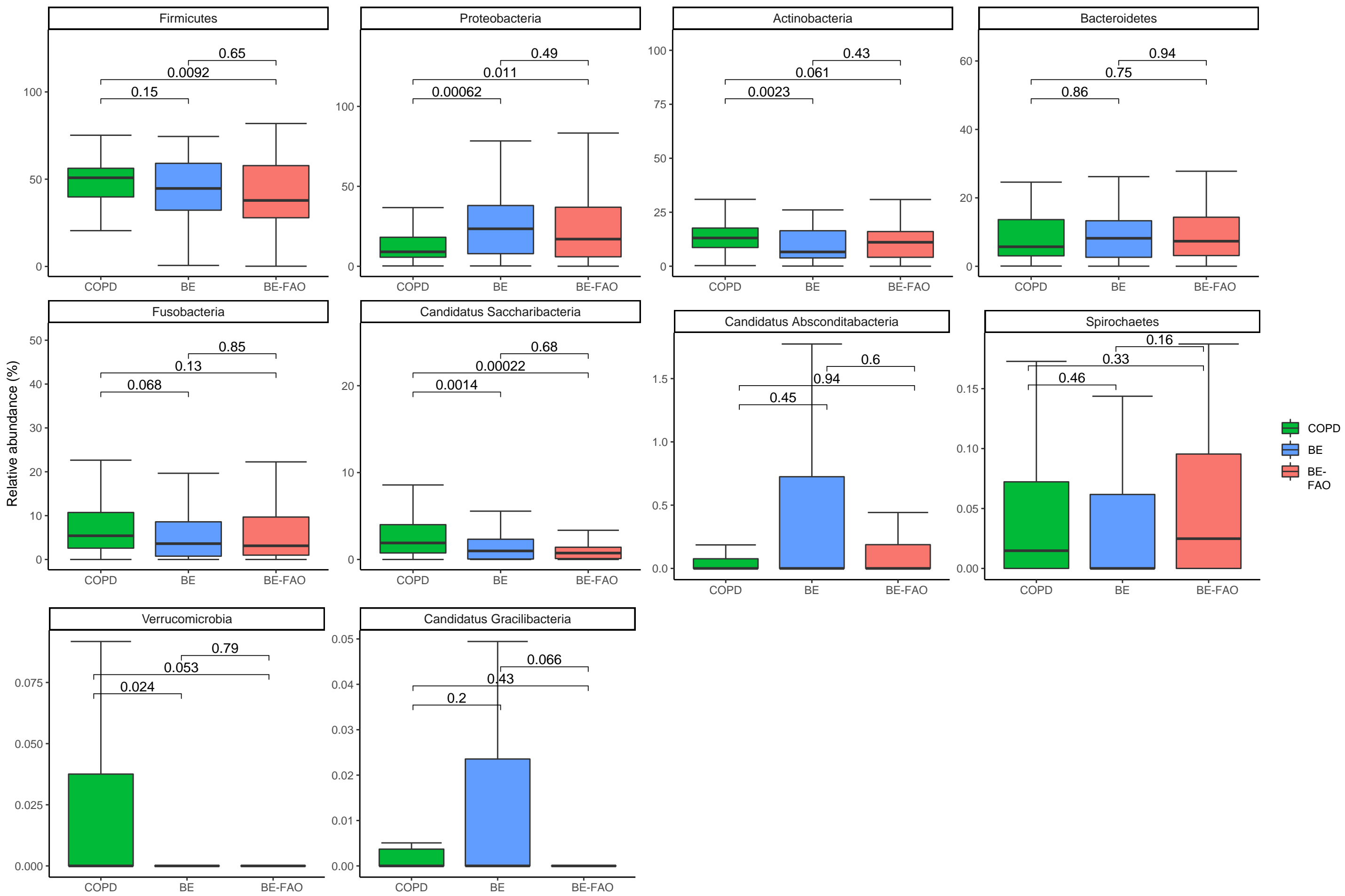

Supplement: Supplementary file 3 — Supplementary Material 3. Figure S3. The distribution of relative abundance of top 10 major taxonomic groups in three groups at phylum level. The patients with BE-FAO had a higher relative abundance of Proteobacteria (p=0.011) and lower abundance of Firmicutes (p=0.0092) relative to the patients with COPD. No significant difference was observed in the proportions of the four major phyla in BE and BE-FAO. BE=Bronchiectasis without fixed airflow obstruction; BE-FAO= Bronchiectasis with fixed airflow obstruction; COPD=Chronic obstructive pulmonary disease. [file 12931_2024_2931_MOESM3_ESM.pdf]

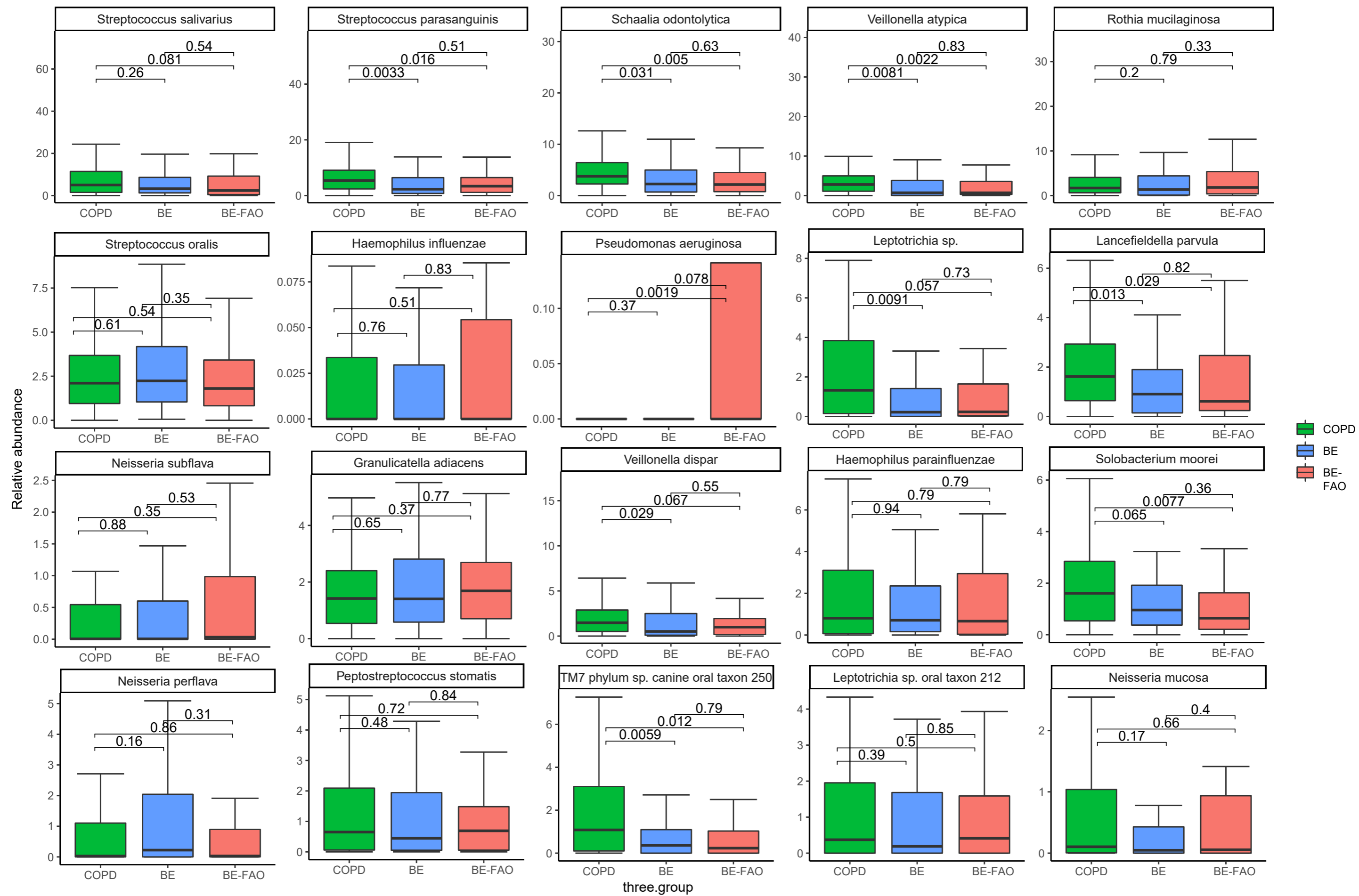

Supplement: Supplementary file 4 — Supplementary Material 4. Figure S4. Highlights species-level taxonomic distribution differences between COPD, BE, and BE-FAO patients. Among ASV annotated to specie, the COPD group showed higher prevalence of Streptococcus parasanguinis, Schaalia odontolytica, Veillonella atypica, Lancefieldella parvula, Solobacterium moorei, and TM7 phylum sp canine oral taxon 250, while Pseudomonas aeruginosa was more abundant in the BE-FAO group. Wilcoxon rank-sum test was used to compare the relative abundance of taxa. BE=Bronchiectasis without fixed airflow obstruction; BE-FAO= Bronchiectasis with fixed airflow obstruction; COPD=Chronic obstructive pulmonary disease. [file 12931_2024_2931_MOESM4_ESM.pdf]

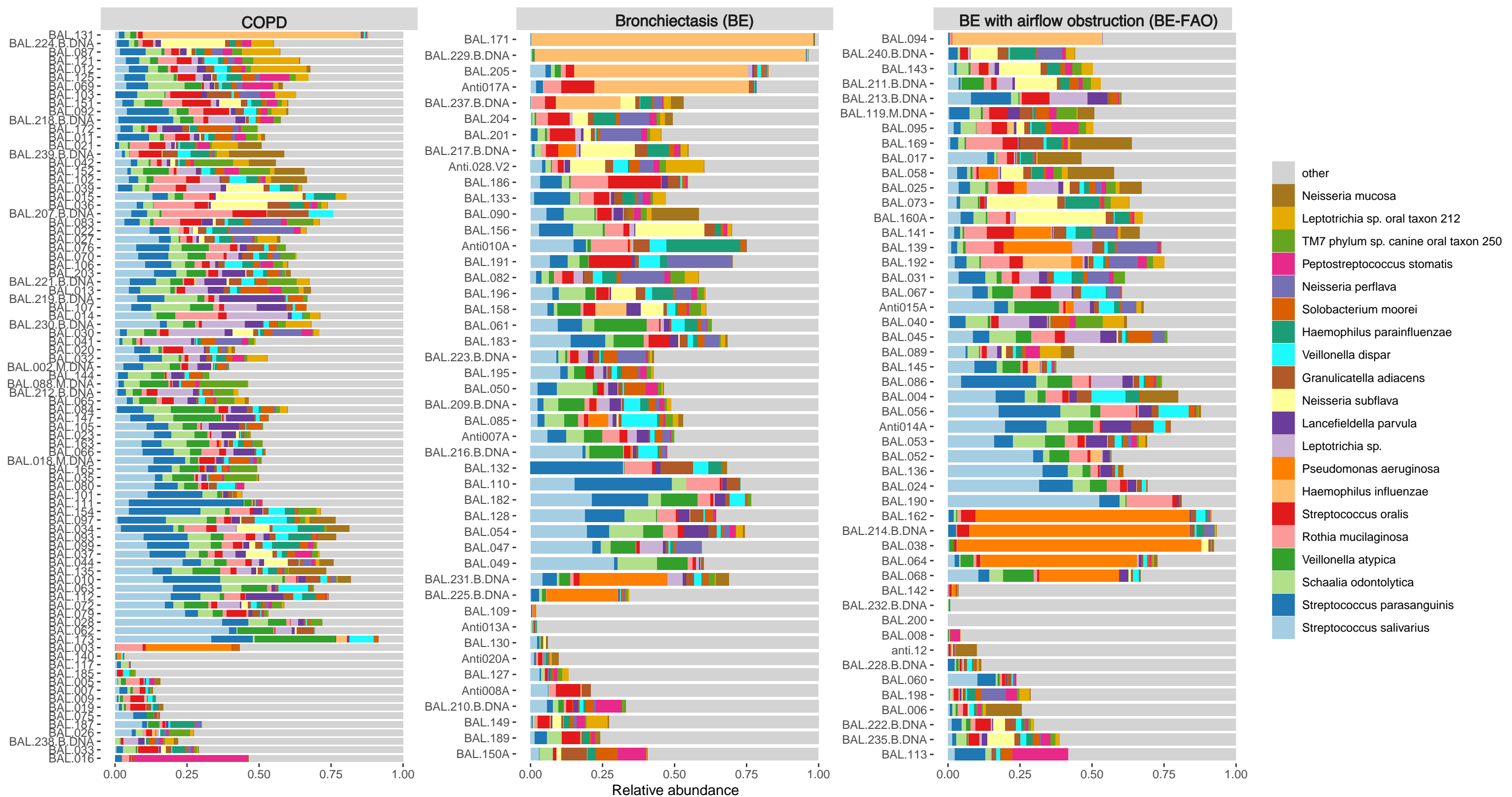

Supplement: Supplementary file 5 — Supplementary Material 5. Figure S5. Stacked plot of relative abundance of taxa at the species level in each sample within COPD (n=86), BE (n=46) and BE-FAO (n=49) group. BE=Bronchiectasis without fixed airflow obstruction; BE-FAO= Bronchiectasis with fixed airflow obstruction; COPD=Chronic obstructive pulmonary disease. [file 12931_2024_2931_MOESM5_ESM.pdf]

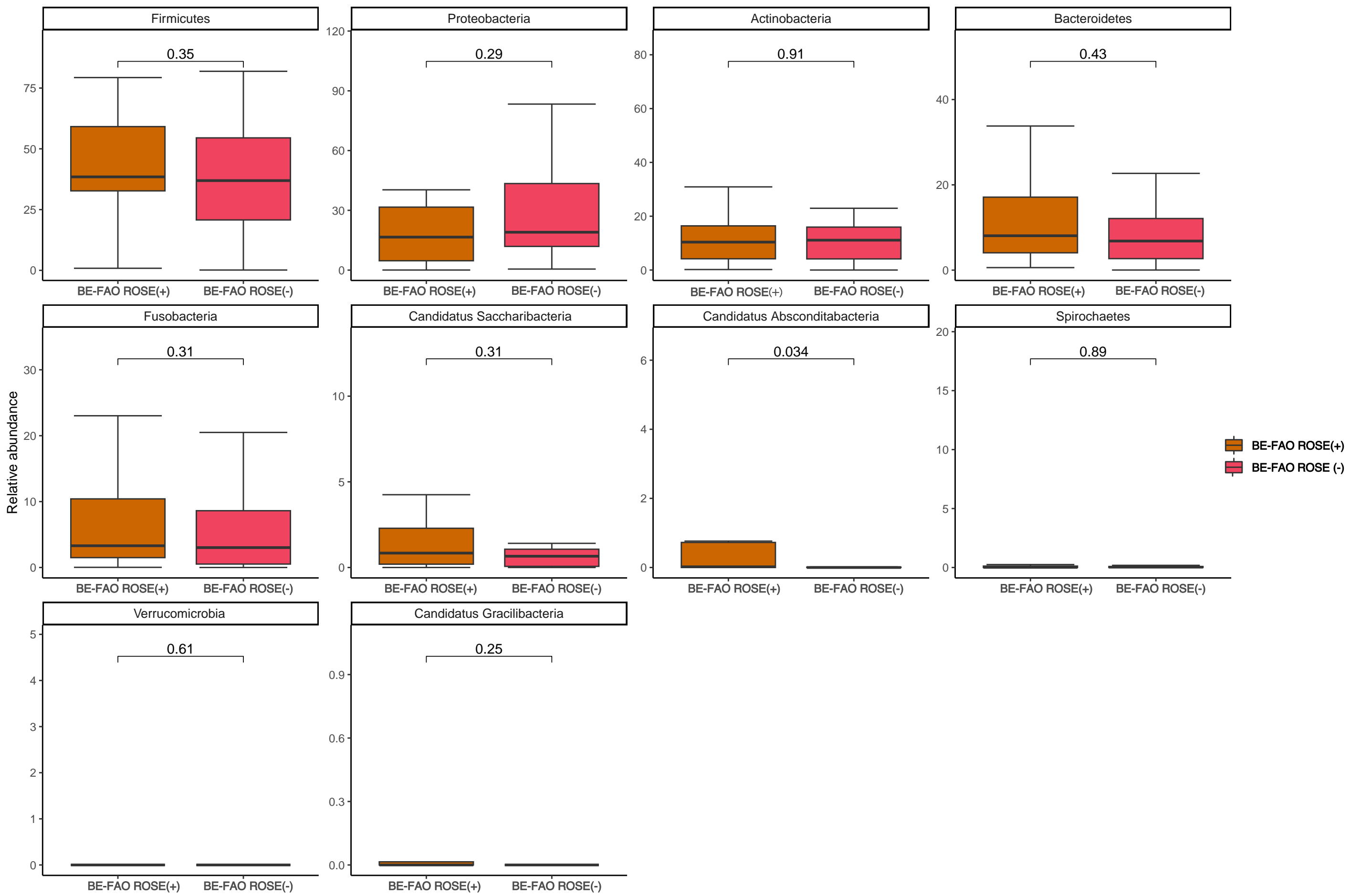

Supplement: Supplementary file 6 — Supplementary Material 6. Figure S6. The difference of lung microbiota composition of patients in BE-FAO ROSE (+) (n=24) and BE-FAO ROSE (−) (n=25). The composition of major taxonomic groups and the distribution of relative abundance of phylum level. The patients with BE-FAO ROSE (+) had a higher relative abundance of Candidatus Absconditabacteria (P=0.034) at the phyla level compared to those with BE-FAO ROSE (−). BE-FAO= Bronchiectasis with fixed airflow obstruction; ROSE=Radiology, Obstruction, Symptoms, Exposure. [file 12931_2024_2931_MOESM6_ESM.pdf]

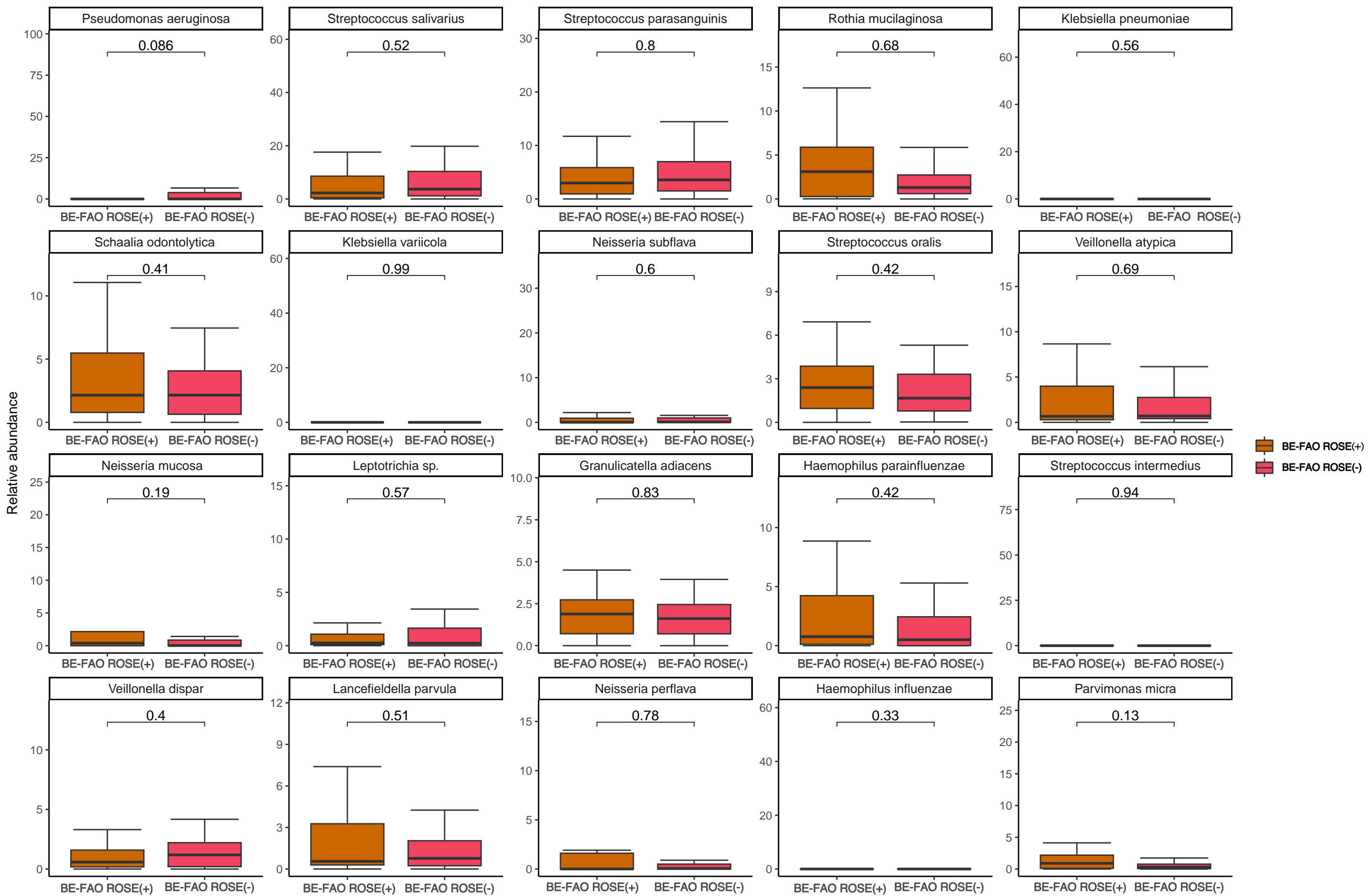

Supplement: Supplementary file 7 — Supplementary Material 7. Figure S7. The difference of lung microbiota composition of patients in BE-FAO ROSE (+) (n=24) and BE-FAO ROSE (−) (n=25). The composition of major taxonomic groups and the distribution of relative abundance of species level. The patients with BE-FAO ROSE (−) had a relative abundance of Pseudomonas aeruginosa (P=0.086) when ASV annotated to species level, compared with those BE-FAO ROSE (+). BE-FAO= Bronchiectasis with fixed airflow obstruction; ROSE=Radiology, Obstruction, Symptoms, Exposure. [file 12931_2024_2931_MOESM7_ESM.pdf]

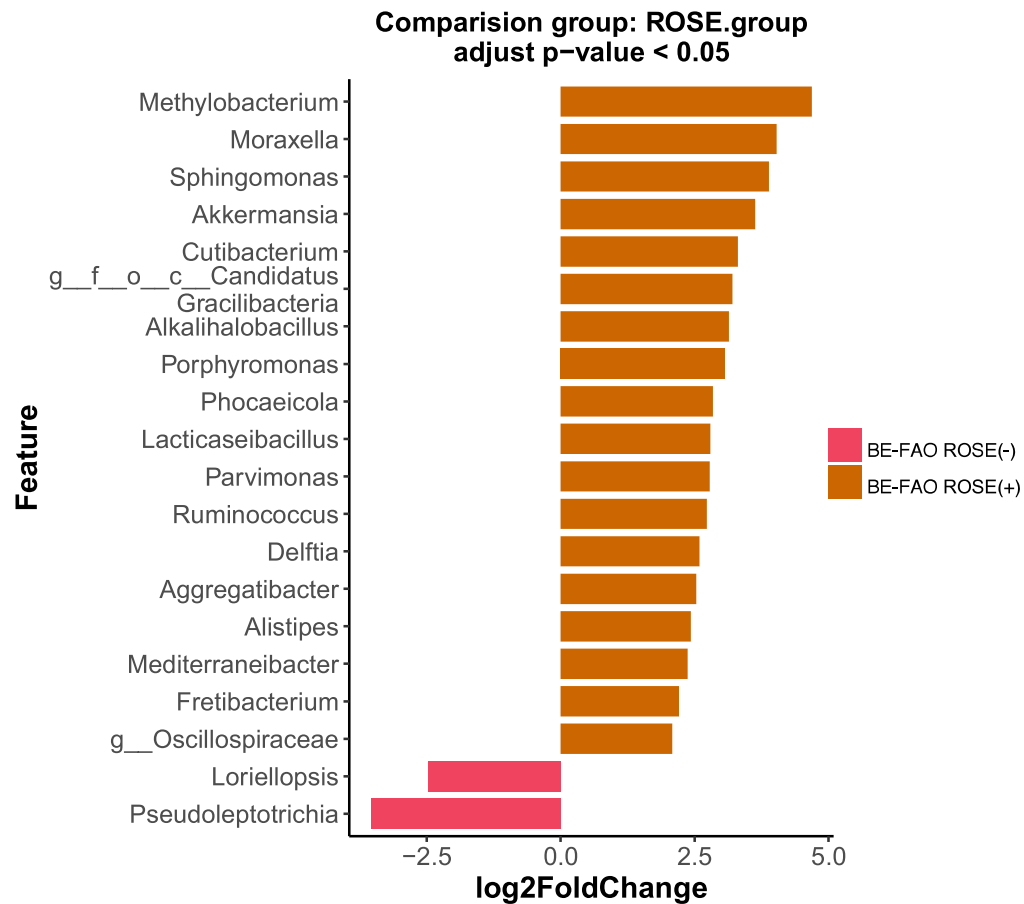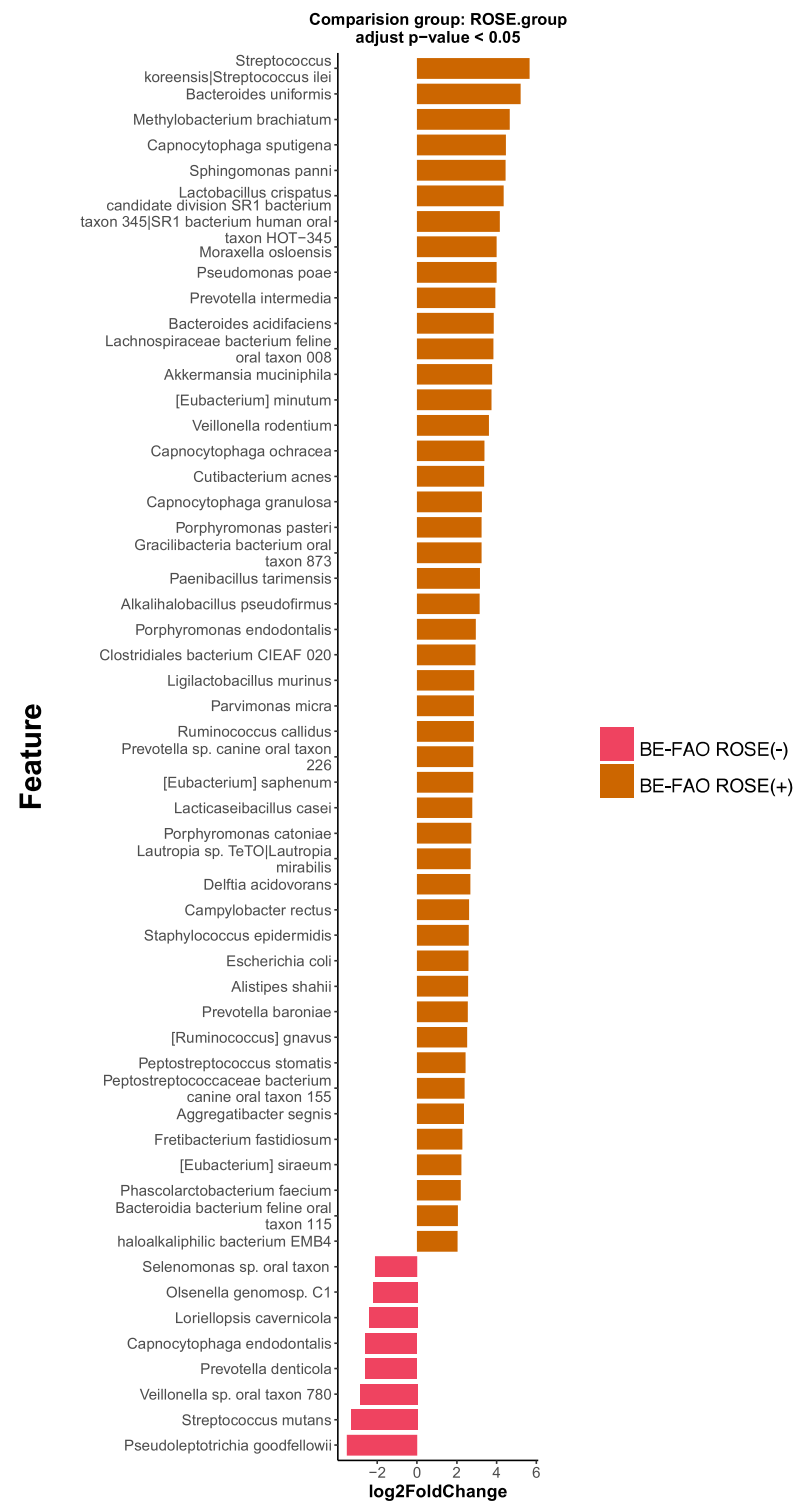

Supplement: Supplementary file 8 — Supplementary Material 8. Figure S8. The differential abundance of lung microbiome analysis using DEseq2 (after adjusting for age and gender) in the BE-FAO group. The different taxonomic levels (adjusted p<0.05 and fold change>2.0) in BE-FAO ROSE (+) versus BE-FAO ROSE (−) at (A) genus level (B) species level. We further disclosed that Pseudoleptotrichia goodfellowii, Streptococcus mutans, Veillonella sp.oral taxon 780, Prevotella denticola, Capnocytophaga endodontalis, Loriellopsis cavernicola, Olsenella genomosp.C1 and Selenomonas sp. oral taxon were enriched in BE-FAO ROSE (−) group compared to BE-FAO ROSE (+) group. BE-FAO= Bronchiectasis with fixed airflow obstruction; ROSE=Radiology, Obstruction, Symptoms, Exposure. [file 12931_2024_2931_MOESM8_ESM.pdf]

**A**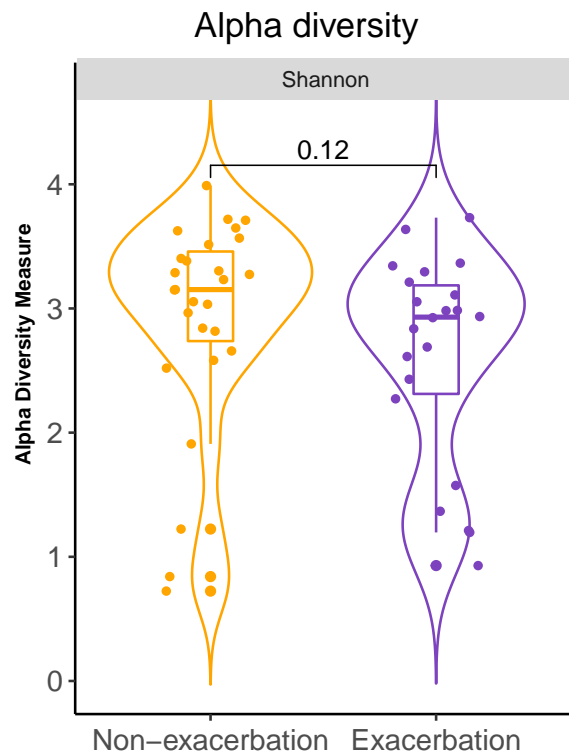**B**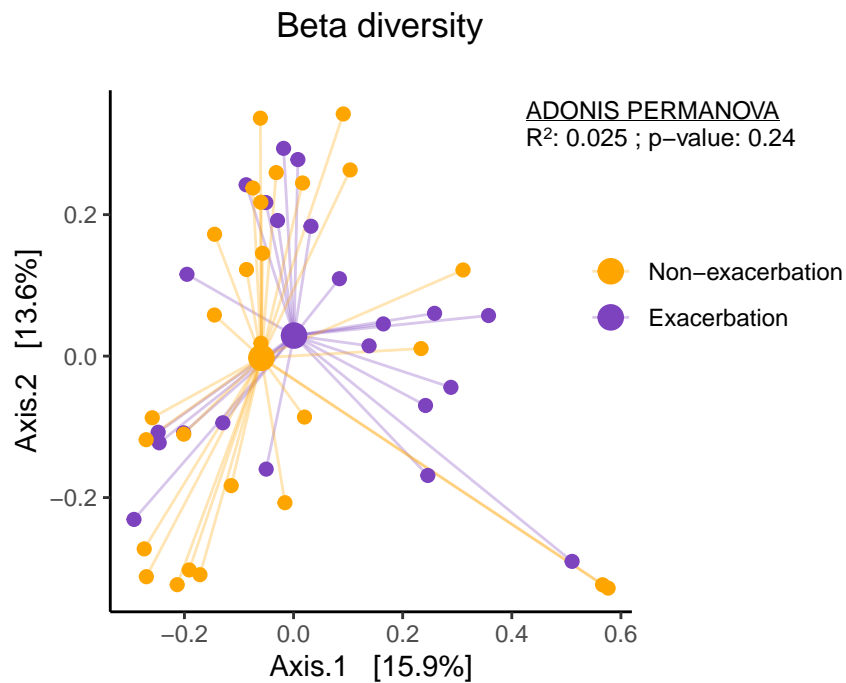

Supplement: Supplementary file 9 — Supplementary Material 9. Figure S9 shows alpha (A) and beta (B) diversity in BE-FAO patients with future exacerbations (n=22) versus those without (n=27) using BAL microbiome profiles. Both alpha diversity (P = 0.12) and beta diversity (R2 = 0.025, P = 0.24) measures were similar between exacerbation and non-exacerbation subgroups. BE-FAO= Bronchiectasis with fixed airflow obstruction. [file 12931_2024_2931_MOESM9_ESM.pdf]

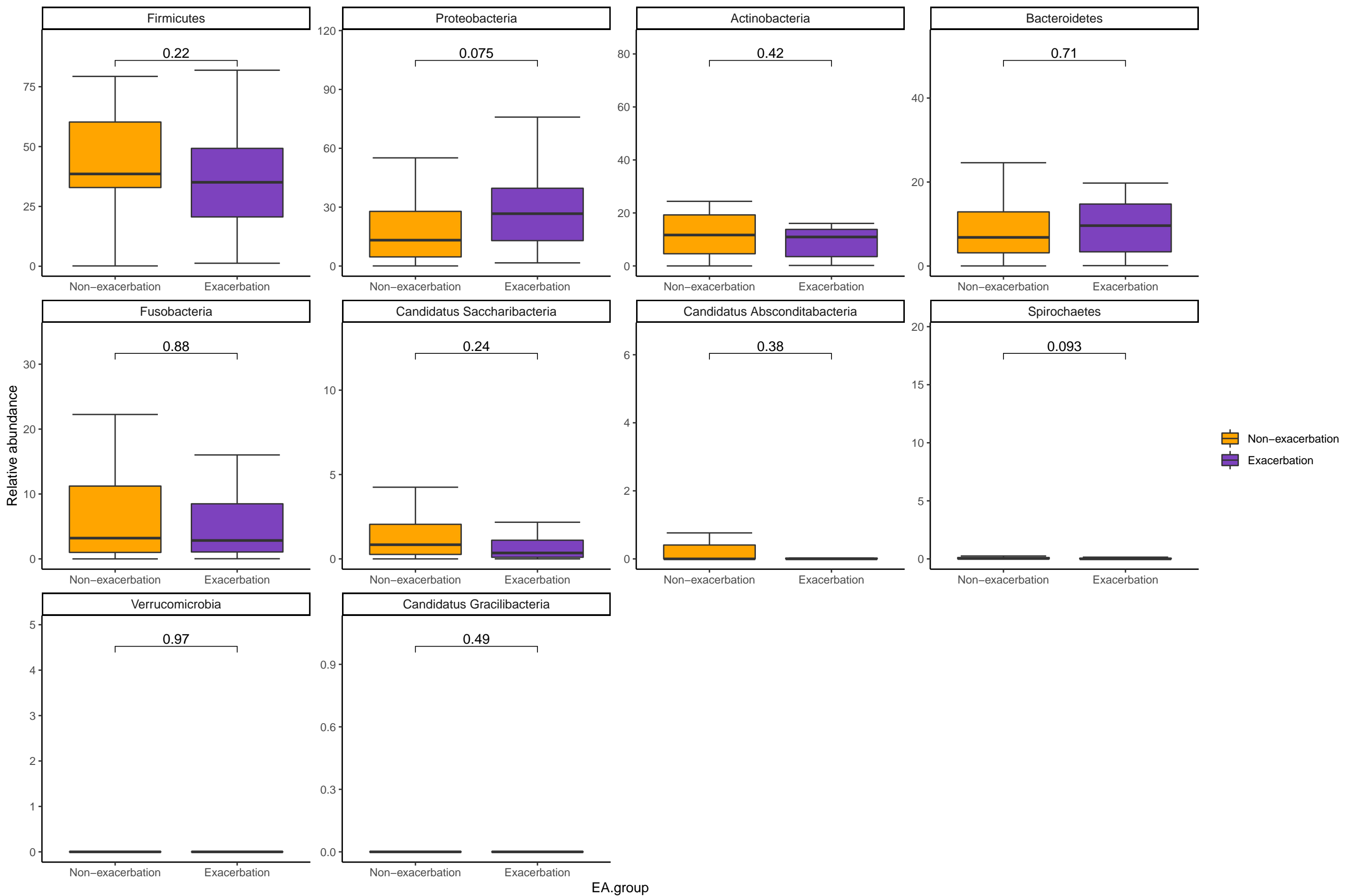

Supplement: Supplementary file 10 — Supplementary Material 10. Figure S10. Differences in lung microbiota composition at the phylum level between patients with exacerbations (n = 22) and non-exacerbations (n = 27) in the BE-FAO group. In this group, the exacerbation subgroup had a higher relative abundance of Proteobacteria (P =0.075) compared with the non-exacerbation subgroup, although this difference was nonsignificant. No significant differences were obtained in other major phyla between the exacerbation and non-exacerbation subgroups. BE-FAO = bronchiectasis with FAO. [file 12931_2024_2931_MOESM10_ESM.pdf]

A

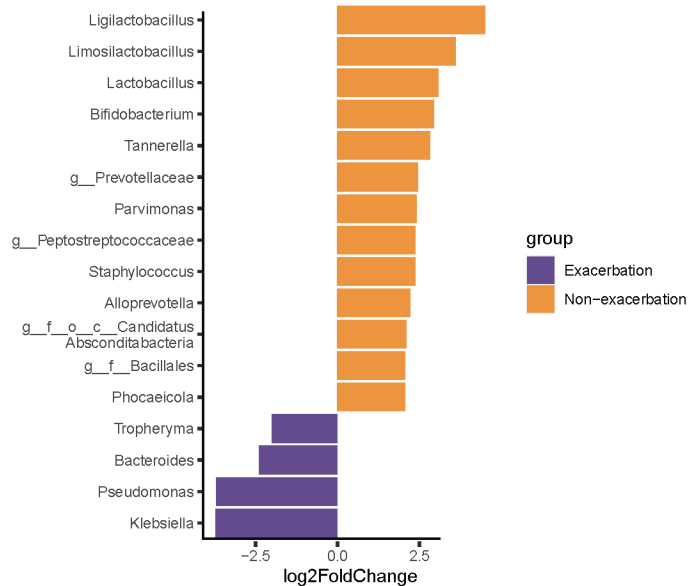

B

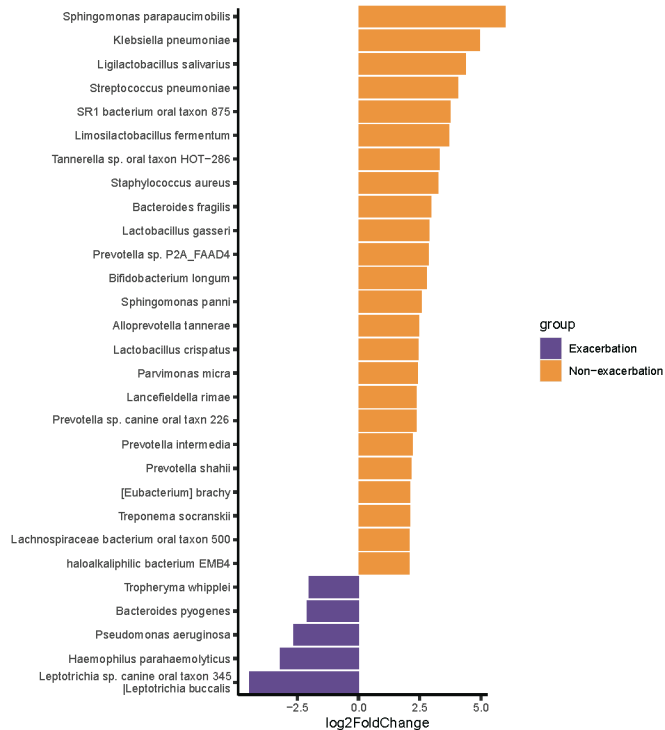

Supplement: Supplementary file 11 — Supplementary Material 11. Figure S11. The differential abundance of lung microbiome analysis using DEseq2 in the BE-FAO group. The different taxonomic levels (adjusted p<0.05 and fold change>2.0) in exacerbation versus non-exacerbation subgroups at (A) phylum level (B) species level. DESeq2 analysis revealed that the exacerbation subgroup of BE-FAO had a predominance of Leptotrichia sp. canine oral taxon 345, Haemophilus parahaemolyticus, Pseudomonas aeruginosa, Bacteroides pyogenes, and Tropheryma whipplei relative to the non-exacerbation subgroup. BE-FAO= Bronchiectasis with fixed airflow obstruction. [file 12931_2024_2931_MOESM11_ESM.pdf]
