# Supplementary material for: The clinical impacts of lung microbiome in bronchiectasis with fixed airflow obstruction: a prospective cohort study
Source: Respir Res. 2024 Aug 14;25:308. doi: 10.1186/s12931-024-02931-x (PMC11325704; doi:10.1186/s12931-024-02931-x)
Supplement: Supplementary file 12 — Supplementary Material 12. [file 12931_2024_2931_MOESM12_ESM.docx]

| **Table S1. Decomtam flow and sequencing data^a,b^** | | | | | | |
| --- | --- | --- | --- | --- | --- | --- |
| **Samples** | **No. ASVs** | **No. Species** | **No. Species of Contaminant** | **No. Species after decontam** | **No. Species after rarefaction** | **No. Species after filter out taxon with prevalence < 10%** |
| **181 BAL** | 7771 | 1750 | 65 | 1685 | 1624 | 295 |
| **78 OWC** | 3951 | 820 | 20 | 800 | - | - |
| **28 NC** | 1378 | 792 | - | - | - | - |

a. The detailed feature of taxon data of BAL OWC and NC samples were provided in **Additional file table S6**

b. Raw sequencing data upload to NCBI SRA --**Project ID: PRJNA924101**

[**https://dataview.ncbi.nlm.nih.gov/object/PRJNA924101?reviewer=6aivli35eho5jdrfoatvvpbf10**](https://dataview.ncbi.nlm.nih.gov/object/PRJNA924101?reviewer=6aivli35eho5jdrfoatvvpbf10)

ASV= Amplicon sequence variants (ASVs); BAL= Bronchoalveolar lavage; NC=Negative control; OWC=oral washing control.
