# Supplementary material for: The clinical impacts of lung microbiome in bronchiectasis with fixed airflow obstruction: a prospective cohort study
Source: Respir Res. 2024 Aug 14;25:308. doi: 10.1186/s12931-024-02931-x (PMC11325704; doi:10.1186/s12931-024-02931-x)
Supplement: Supplementary file 13 — Supplementary Material 13. [file 12931_2024_2931_MOESM13_ESM.docx]

| **Table S2 The comparison of conventional culture and 16S rRNA gene sequencing results** | | | | |
| --- | --- | --- | --- | --- |
| **Taxa name** | **Conventional Culture** | **16S rRNA sequencing** | | |
|  | n | n | Mean relative abundance (%) | 16S sequencing detection rate (%) (compared to culture) |
| **Species** | | | | |
| *Klebsiella pneumoniae* | 57 | 39 | 6.912 | 68.4 |
| *Staphylococcus aureus* | 32 | 20 | 1.899 | 62.5 |
| *Pseudomonas aeruginosa* | 30 | 23 | 17.859 | 76.7 |
| *Haemophilus influenzae* | 16 | 15 | 32.241 | 93.8 |
| *Haemophilus parahaemolyticus* | 10 | 8 | 1.299 | 80.0 |
| *Haemophilus parainfluenzae* | 10 | 10 | 2.448 | 100.0 |
| *Escherichia coli* | 7 | 7 | 24.238 | 100.0 |
| *Mycobacterium chimaera intracellulare group* | 7 | 0 | NA | 0.0 |
| *Enterobacter cloacae complex* | 4 | 0 | NA | 0.0 |
| *Klebsiella oxytoca* | 4 | 0 | NA | 0.0 |
| *Stenotrophomonas maltophilia* | 4 | 1 | 0.244 | 25.0 |
| *Streptococcus agalactiae* | 4 | 3 | 0.023 | 75.0 |
| *Streptococcus pneumoniae* | 4 | 2 | 3.547 | 50.0 |
| *Klebsiella variicola* | 3 | 1 | 0.324 | 33.3 |
| *Acinetobacter pittii* | 2 | 0 | NA | 0.0 |
| *Haemophilus haemolyticus* | 2 | 1 | 0.027 | 50.0 |
| *Serratia marcescens* | 2 | 0 | NA | 0.0 |
| *Streptococcus anginosus* | 2 | 2 | 1.004 | 100.0 |
| *Acinetobacter baumannii* | 1 | 0 | NA | 0.0 |
| *Acinetobacter junii* | 1 | 0 | NA | 0.0 |
| *Alcaligenes faecalis* | 1 | 0 | NA | 0.0 |
| *Beta-streptococcus* | 1 | 0 | NA | 0.0 |
| *Bordetella sp.* | 1 | 0 | NA | 0.0 |
| *Chryseobacterium gleum* | 1 | 0 | NA | 0.0 |
| *Comamonas terrigena* | 1 | 0 | NA | 0.0 |
| *Haemophilus paraphrohaemolyticus* | 1 | 0 | NA | 0.0 |
| *Klebsiella aerogenes* | 1 | 0 | NA | 0.0 |
| *Moraxella_sg_Branhamella catarrhalis* | 1 | 1 | 38.161 | 100.0 |
| *Mycobacterium colombiense* | 1 | 0 | NA | 0.0 |
| *Mycobacterium interjectum* | 1 | 0 | NA | 0.0 |
| *Neisseria gonorrhoeae* | 1 | 1 | 17.283 | 100.0 |
| *Pasteurella multocida* | 1 | 1 | 28.508 | 100.0 |
| *Proteus vulgaris* | 1 | 0 | NA | 0.0 |
| *Raoultella ornithinolytica* | 1 | 0 | NA | 0.0 |
| *Streptococcus constellatus* | 1 | 1 | 0.618 | 100.0 |
| *Streptococcus dysgalactiae* | 1 | 1 | 0.203 | 100.0 |
| *Wautersiella falsenii* | 1 | 0 | NA | 0.0 |
|  | **211** | **137** |  | **64.9%** |
| **Genus** | | | | |
| *Mycobacterium (Mycobacterium chimaera intracellulare group, Mycobacterium species(NTM),Mycobacterium avium intracellulare complex,Mycobacterium species, Mycobacterium tuberculosis complex)* | 6 | 2 | 5.905 | 33.3 |
| *Nocardia species* | 3 | 2 | 4.938 | 66.7 |
| *Acinetobacter species* | 2 | 2 | 1.584 | 100.0 |
| *Achromobacter species* | 1 | 1 | 1.635 | 100.0 |
| *Aeromonas species* | 1 | 0 | 0.000 | 0.0 |
| *Cunninghamella species* | 1 | 0 | 0.000 | 0.0 |
| *Pseudomonas species* | 1 | 1 | 0.029 | 100.0 |
| *Rhizopus species* | 1 | 0 | 0.000 | 0.0 |
|  | **21** | **14** |  | **66.7%** |
