# Supplementary material for: The clinical impacts of lung microbiome in bronchiectasis with fixed airflow obstruction: a prospective cohort study
Source: Respir Res. 2024 Aug 14;25:308. doi: 10.1186/s12931-024-02931-x (PMC11325704; doi:10.1186/s12931-024-02931-x)
Supplement: Supplementary file 14 — Supplementary Material 14. [file 12931_2024_2931_MOESM14_ESM.docx]

| **Table S3 Clinical variables and outcomes of patients with bronchiectasis with FAO** | | | | |
| --- | --- | --- | --- | --- |
|  | **BE with FAO (n=49)** | | | |
| **Clinical factors/variables** | **ROSE (+)** | **ROSE (-)** | P value | |
| **Number** | **24** | **25** |  | |
| **Age, years, median (IQR)** | 76.7(69.7-79.8) | 70.1(58.0-76.4) | 0.026* | |
| **Gender, Man, n(%)** | 24(100) | 11(44.0) | <0.001* | |
| **BMI (kg/m2), median (IQR)** | 22.4(20.1-25.4) | 20.6(19.5-24.2) | 0.150 | |
| **Smoking status, n (%)** |  |  |  | |
| Nonsmoker | 0(0) | 25(100) | <0.001* | |
| Current smoker or ex-smoker | 24(100) | 0(0) |  |  |
| **Smoking pack-years, median (IQR)** | 41.5(10.0-75.0) | 0(0) | <0.001* | |
| **Etiologies of Bronchiectasis** |  |  |  | |
| Idiopathic | 0 (0) | 12(48.0) | <0.001* | |
| Post-infection |  |  |  |  |
| Pneumonia | 5(20.8) | 3(12.0) |  |  |
| NTM or TB | 6 (25.0) | 10 (40.0) |  |  |
| COPD | 13(54.2) | 0(0) |  |  |
| **History of tuberculosis infection, n(%)** | 6(25.0) | 8(32.0) | 0.411 | |
| **Lung function test, median (IQR)** |  |  |  | |
| FEV1/FVC (%) | 63.0(49.5-66.5) | 64.6(61.2-68.8) | 0.147 | |
| FEV1 (%) | 70.0(42.2-80.9) | 69.2(58.4-79.8) | 0.555 | |
| FVC (%) | 85.6(74.7-105.7) | 88.6(73.7-97.9) | 0.841 | |
| Bronchodilator reversibility, n (%) | 5(20.8) | 1(4.0) | 0.086 | |
| **Radiological severity of Bronchiectasis** |  |  |  | |
| Bronchiectasis involved lobes, median (IQR) | 4.0(3.0-5.0) | 5.0(3.0-5.5) | 0.610 | |
| Modified Reiff score, median (IQR) | 4.0(3.0-6.0) | 5.0(3.0-7.0) | 0.419 | |
| **Bronchiectasis severity index (BSI), median (range)** | 9.0(7.0-10.0) | 8.0(5.5-9.5) | 0.231 | |
| Mild (0-4), n(%) | 2(8.3) | 4(16.0) | 0.538 | |
| Moderate (5-8), n(%) | 9(37.5) | 11(44.0) |  |  |
| Severe (>9), n(%) | 13(54.2) | 10(40.0) |  |  |
| **Emphysema LAV (%), median (IQR)** | 11.6(5.6-22.9) | 3.2(2.0-5.1) | <0.001* | |
| **mMRC (dyspnea scale), n (%)** |  |  |  | |
| 0-1 | 8(33.3) | 16(64.0) | 0.031* | |
| > 2 | 16(66.7) | 9(36.0) |  |  |
| **CAT score (symptoms score), n(%)** |  |  |  | |
| <10 | 16(66.7) | 19(76.0) | 0.342 | |
| >10 | 8(33.3) | 6(24.0) |  |  |
| **Exacerbation in the prior year, n(%)** |  |  |  | |
| High risk, >2 times/year | 18(75.0) | 20(75) | 0.469 | |
| Low risk, 0-1 time/year | 6(25.0) | 5(20.0) |  |  |
| **Inhalation therapy, n (%) at baseline** |  |  |  | |
| Short-acting bronchodilator or none | 4(16.7) | 6(24.0) | 0.886 | |
| Monotherapy (LAMA or LABA) | 2(8.3) | 2(8.0) |  |  |
| Dual bronchodilators (LAMA+LABA) | 14(58.3) | 12(48.0) |  |  |
| Triple therapy | 4(16.7) | 5(20.0) |  |  |
| Inhaled corticosteroid (ICS) | 4(16.7) | 5(20.0) | 0.527 | |
| **Clinical outcomes** |  |  |  | |
| Moderate or severe exacerbation | 10(41.0) | 12(48.0) | 0.437 | |
| Severe exacerbation | 9(37.5) | 5(20.0) | 0.149 | |
| Data are presented as No. (%) or median (interquartile range), unless otherwise indicated; n.a.: not available | | | |  |

For each row, data are either % with p-values from t test or Fisher’s exact tests between the two groups, median (interquartile range) with p-values from Mann‐Whitney tests; *p <0.05. BE = Bronchiectasis without fixed airflow obstruction; BE-FAO = bronchiectasis with fixed airflow obstruction; BMI = Body Mass Index; COPD = Chronic obstructive pulmonary disease; ROSE=Radiology, Obstruction, Symptoms, Exposure;FEV1 = forced expiratory volume in 1 sec; FVC = forced vital capacity; LAV = low-attenuation volume; CAT = COPD Assessment Test; mMRC = modified Medical Research Council; LAMA = long-acting muscarinic antagonist; LABA = Long-acting β2 Sympathomimetic Agonists; NTM=Non-tuberculosis mycobacteria; ICS = Inhaled corticosteroid; TB=Tuberculosis.
