# Supplementary material for: The clinical impacts of lung microbiome in bronchiectasis with fixed airflow obstruction: a prospective cohort study
Source: Respir Res. 2024 Aug 14;25:308. doi: 10.1186/s12931-024-02931-x (PMC11325704; doi:10.1186/s12931-024-02931-x)
Supplement: Supplementary file 15 — Supplementary Material 15. [file 12931_2024_2931_MOESM15_ESM.docx]

| **Table S4. Laboratory data and BAL culture of patients with bronchiectasis with FAO** | | | |  |
| --- | --- | --- | --- | --- |
|  | **BE with FAO (n=49)** | | |  |
| **Laboratory data** | **ROSE (+) (n=24)** | **ROSE (-) (n=25)** | P value |  |
| **Blood sample, median (IQR)** |  |  |  |  |
| Hemoglobin (g/dl) | 14.2(13.0-14.8) | 13.6(12.4-14.8) | 0.432 |  |
| Platelet count (K/μl) | 222.0(175.7-270.2) | 250.0(219.0-290.5) | 0.119 |  |
| White blood cell counts (K cells/mm^3^) | 7.55(5.43-9.14) | 7.51(5.41-9.35) | 0.810 |  |
| Neutrophil (%) | 65.9(56.5-69.7) | 63.2(57.9-73.1) | 0.849 |  |
| Eosinophil (%) | 3.2(1.8-4.6) | 1.9(0.9-2.7) | 0.017* |  |
| <2 %, n (%) | 6(25.0) | 13(52.0) | 0.049* |  |
| >2%, n (%) | 18(75.0) | 12(40.0) |  |  |
| Eosinophil counts | 225.7(137.1-303.7) | 149.4(81.6-206.62) | 0.050 |  |
| Lymphocyte (%) | 22.5(18.1-31.0) | 28.1(19.0-33.8) | 0.317 |  |
| Monocyte (%) | 6.4(5.9-7.4) | 6.2(4.9-7.2) | 0.267 |  |
| C-reactive protein (mg/dL) | 0.37(0.15-0.89) | 0.45(0.27-0.88) | 0.529 |  |
| **BAL samples, median (IQR)** |  |  |  |  |
| Macrophage % | 86.8(79.2-90.8) | 85.1(82.0-93.1) | 0.522 |  |
| Neutrophils % | 2.1(0.9-3.5) | 2.7(0.8-8.4) | 0.390 |  |
| Eosinophils % | 1.8(1.1-2.6) | 1.8(1.3-3.0) | 0.459 |  |
| Lymphocyte % | 9.3(5.4-14.7) | 4.6(2.7-9.2) | 0.016* |  |
| **Broncholavage sample, median (IQR)** |  |  |  |  |
| Eotaxin (pg/ml) | 1.9(0.9-4.9) | 1.9(0.9-3.1) | 0.653 |  |
| IL-1β (pg/ml) | 8.2(3.4-141.2) | 230.9(21.7-651.5) | 0.016* |  |
| IL-6 (pg/ml) | 15.6(2.9-33.4) | 46.5(16.4-109.4) | 0.004* |  |
| IL-18 (pg/ml) | 35.1(21.22-50.07) | 41.1(23.8-48.9) | 0.298 |  |
| IL-8 (pg/ml) | 309.8(164.7-2525.2) | 1288.3(404.0-2689.4) | 0.072 |  |
| TNF-α (pg/ml) | 9.8(5.0-20.9) | 31.2(8.0-75.9) | 0.050 |  |
| MCP-1(pg/ml) | 199.2(103.5-411.2) | 428.3(237.3-712.5) | 0.007* |  |
| NETs (pg/ml) | 0.8(0.3-2.6) | 1.3(0.6-2.9) | 0.201 |  |
| **Conventional culture of BAL samples** |  |  |  |  |
| *Klebsiella pneumoniae, n (%)* | 12(50.0) | 6(24.0) | 0.055 |  |
| *Pseudomonas aeroginosa, n (%)* | 6(25.0) | 10(40.0) | 0.208 |  |
| *Staphylococcus aureus, n (%)* | 4(16.7) | 7(28.0) | 0.273 |  |
| *Haemophilus influenzae, n (%)* | 1(4.2) | 2(8.0) | 0.516 |  |
| Non-tuberculosis mycobacterium, n (%) | 1(4.2) | 6(24.0) | 0.055 |  |
| *Asepergillus* spp, n (%) | 0(0) | 5(20.0) | 0.028* |  |
| *Candida* spp, | 7(29.2) | 5(20.0) | 0.340 |  |
| Potential pathogenic bacteria colonization, n (%) | 21(87.5) | 23(92.0) | 0.480 |  |
| Data are presented as No. (%) or median (interquartile range), unless otherwise indicated | | | |  |

For each row, data are either % with p-values from t test or Fisher’s exact tests between the two groups, median (IQR) with p-values from Mann‐Whitney tests; *p <0.05. BAL=Bronchoalveolar lavage; BE=Bronchiectasis without fixed airflow obstruction; BE-FAO=Bronchiectasis with fixed airflow obstruction; ROSE=Radiology, Obstruction, Symptoms, Exposure; CRP=C-reactive protein; IL-1β=interleukin [IL]-1β; IL-6=interleukin [IL]-6; IL-8= interleukin [IL]-8; IL-18=interleukin [IL]-18; MCP-1=Monocyte chemoattractant protein-1; NETs= Neutrophil extracellular traps; TNF-α=tumor necrosis factor [TNF]-α. R
