# Supplementary material for: The clinical impacts of lung microbiome in bronchiectasis with fixed airflow obstruction: a prospective cohort study
Source: Respir Res. 2024 Aug 14;25:308. doi: 10.1186/s12931-024-02931-x (PMC11325704; doi:10.1186/s12931-024-02931-x)
Supplement: Supplementary file 16 — Supplementary Material 16. [file 12931_2024_2931_MOESM16_ESM.docx]

| **Table S5 Clinical variables of patients with bronchiectasis with FAO** | | | |  |
| --- | --- | --- | --- | --- |
|  | **BE with FAO (n=49)** | | |  |
| **Clinical factors/variables** | **Non-exacerbation** | **Exacerbation** | *P* value |  |
| **Number** | **27** | **22** |  |  |
| **Age, years, median (IQR)** | 73.6(61.5-79.1) | 73.4(63.8-78.9) | 0.968 |  |
| **Gender, Man, n(%)** | 21(77.8) | 14(63.3) | 0.220 |  |
| **BMI (kg/m^2^), median (IQR)** | 22.3(19.9-25.4) | 21.6(19.4-23.5) | 0.335 |  |
| **Smoking status, n (%)** |  |  |  |  |
| Nonsmoker | 13 (48.1) | 12(54.5) | 0.437 |  |
| Current smoker or ex-smoker | 14(51.8) | 10(45.5) |  |  |
| **Classification of BE-FAO** |  |  |  |  |
| ROSE criteria (+) | 14(51.9) | 10(45.5) | 0.437 |  |
| ROSE criteria (-) | 13 (48.1) | 12(54.5) |  |  |
| **History of tuberculosis infection, n (%)** | 7(25.9) | 7(31.8) | 0.444 |  |
| **Lung function test, median (IQR)** |  |  |  |  |
| FEV_1/_FVC (%) | 62.9(59.7-66.2) | 64.8(57.5-67.5) | 0.494 |  |
| FEV_1_ (%) | 73.5(55.8-83.5) | 64.9(50.4-72.4) | 0.062 |  |
| FVC (%) | 91.1(78.6-108.5) | 80.4(68.9-89.0) | 0.017* |  |
| Bronchodilator reversibility, n (%) | 1(2.7) | 5(22.7) | 0.056 |  |
| **Radiological severity of Bronchiectasis** |  |  |  |  |
| Bronchiectasis involved lobes, median (IQR) | 4.0(3.0-5.0) | 5.0(3.0-6.0) | 0.361 |  |
| Modified Reiff score, median (IQR) | 4.0(3.0-6.0) | 5.0(3.0-6.25) | 0.422 |  |
| **Bronchiectasis severity index (BSI), median (IQR)** | 7.0(5.0-9.0) | 9.0(7.0-10.2) | 0.057 |  |
| **mild (0-4)** | 5(18.5) | 1(4.5) | 0.184 |  |
| **moderate (5-8)** | 12(44.0) | 8(36.4) |  |  |
| **severe (>9)** | 10(37.0) | 13 (59.1) |  |  |
| **Emphysema LAV (%), median (IQR)** | 3.6(2.2-13.4) | 5.6(3.7-17.6) | 0.236 |  |
| **mMRC (dyspnea scale), n (%)** |  |  |  |  |
| 0-1 | 15(55.6) | 9(40.9) | 0.232 |  |
| > 2 | 12(44.0) | 13 (59.1) |  |  |
| **CAT score (symptoms score), n (%)** |  |  |  |  |
| <10 | 17(63.0) | 18(81.8) | 0.128 |  |
| >10 | 10(37.0) | 4(18.2) |  |  |
| **Exacerbation in the prior year, n (%)** |  |  |  |  |
| High risk, >2 times/year | 4(14.8) | 7(31.8) | 0.141 |  |
| Low risk, 0-1 time/year | 23(85.2) | 15(68.2) |  |  |
| **Inhalation therapy, n(%)**  **at baseline** |  |  |  |  |
| Short-acting bronchodilator or none | 7(25.0) | 3(13.6) | 0.750 |  |
| Monotherapy (LAMA or LABA) | 2(7.4) | 2(9.1) |  |  |
| Dual bronchodilators (LAMA+LABA) | 13(48.1) | 13(59.1) |  |  |
| Triple therapy | 5(18.5) | 4(18.2) |  |  |
| Inhaled corticosteroid (ICS) | 5(18.5) | 4(18.2) | 0.635 |  |
| **Blood sample, median (IQR)** |  |  |  |  |
| Hemoglobin (g/dl) | 13.8(12.7-14.9) | 13.8(12.7-14.7) | 1.000 |  |
| Platelet count (K/μl) | 233.0(211.0-287.0) | 254.0(200.2-286.2) | 0.410 |  |
| White blood cell counts (K cells/mm^3^) | 7.21(5.15-8.09) | 7.60(5.42-9.40) | 0.387 |  |
| Neutrophil (%) | 60.6(54.6-66.4) | 69.3(63.1-74.0) | 0.004* |  |
| Eosinophil (%) | 2.8(1.9-4.4) | 1.90(0.9-4.0) | 0.086 |  |
| Eosinophil counts (cells/mm^3^) | 182.9(136.3-289.5) | 146.1(89.6-243.8) | 0.240 |  |
| CRP (mg/dL) | 0.38(0.15-0.77) | 0.73(0.27-1.15) | 0.112 |  |
| **Bronchoalveolar lavage sample, median (IQR)** |  |  |  |  |
| Macrophage (%) | 84.9(77.9-91.2) | 86.9(81.2-92.0) | 0.520 |  |
| Neutrophils (%) | 2.4(1.2-7.7) | 2.1(0.6-4.6) | 0.269 |  |
| Eosinophils (%) | 1.7(1.0-2.5) | 2.1(1.3-4.0) | 0.260 |  |
| Lymphocyte % | 8.4(3.9-10.7) | 7.0(3.7-11.0) | 0.630 |  |
| **Bronchoalveolar lavage sample, mean (SD)** |  |  |  |  |
| Eotaxin (pg/ml) | 2.61(1.10-4.43) | 1.75(0.87-2.44) | 0.215 |  |
| IL-1β (pg/ml) | 39.7(4.02-265.1) | 172.7(7.0-1210.0)) | 0.017* |  |
| IL-6 (pg/ml) | 11.8(5.3-46.5) | 43.4(19.0-105.4) | 0.686 |  |
| IL-18 (pg/ml) | 36.2(23.4-52.5) | 38.8(23.5-47.7) | 0.618 |  |
| IL-8 (pg/ml) | 441.8(214.0-1535.3) | 1557.7(312.5-3003.8) | 0.017* |  |
| TNF-α (pg/ml) | 12.7(4.8-30.1) | 23.4(5.6-86.6) | 0.063 |  |
| MCP-1(pg/ml) | 251.4(118.6-542.4) | 356.3(202.4-578.9) | 0.949 |  |
| NETs (pg/ml) | 0.88(0.35-2.58) | 1.65(0.63-2.95) | 0.301 |  |
| **Conventional culture of BAL samples** |  |  |  |  |
| *Klebsiella pneumoniae,* n (%) | 10(37.0) | 8(36.4) | 0.599 |  |
| *Pseudomonas aeroginosa,* n (%) | 7(25.9) | 9(40.9) | 0.210 |  |
| *Staphylococcus aureus,* n (%) | 6(22.2) | 5(22.7) | 0.616 |  |
| *Haemophilus influenzae,* n (%) | 1(3.7) | 2(9.1) | 0.422 |  |
| Non-tuberculosis mycobacterium, n(%) | 2(7.4) | 5(22.7) | 0.133 |  |
| *Asepergillus* spp, n (%) | 2(7.4) | 3(13.6) | 0.401 |  |
| *Candida* spp, n (%) | 8(29.6) | 4(18.2) | 0.279 |  |
| Potential pathogenic bacteria colonization, n (%) | 23(85.5) | 21(95.5) | 0.245 |  |
| Data are presented as No. (%) or median (interquartile range), unless otherwise indicated; For each row, data are either % with *P*-values from *t* test or Fisher’s exact tests between the two groups, median (IQR) with p-values from Mann‐Whitney tests. BAL=Bronchoalveolar lavage; BE=Bronchiectasis without fixed airflow obstruction; BE-FAO=Bronchiectasis with fixed airflow obstruction; BMI=Body Mass Index; COPD=Chronic obstructive pulmonary disease; ROSE=Radiology, Obstruction, Symptoms, Exposure; FEV1=forced expiratory volume in 1 sec; FVC=forced vital capacity; LAV=low-attenuation volume; HU: Hounsfield unit; CAT=COPD Assessment Test; mMRC=modified Medical Research Council; IL-1β=interleukin [IL]-1β; IL-6=interleukin [IL]-6; IL-8= interleukin [IL]-8; IL-18=interleukin [IL]-18; MCP-1=Monocyte chemoattractant protein-1; NETs= Neutrophil extracellular traps; TNF-α=tumor necrosis factor [TNF]-α. | | | |  |
